# Maternal SMCHD1 regulates *Hox* gene expression and patterning in the mouse embryo

**DOI:** 10.1038/s41467-022-32057-x

**Published:** 2022-07-25

**Authors:** Natalia Benetti, Quentin Gouil, Andres Tapia del Fierro, Tamara Beck, Kelsey Breslin, Andrew Keniry, Edwina McGlinn, Marnie E. Blewitt

**Affiliations:** 1 The Epigenetics and Development Division, WEHI, Parkville, VIC Australia; 2 The Department of Medical Biology, The University of Melbourne, Parkville, VIC Australia; 3 EMBL Australia, Monash University, Clayton, VIC Australia; 4 Australian Regenerative Medicine Institute, Monash University, Clayton, VIC Australia

**Keywords:** Epigenetic memory, Pattern formation, Epigenomics

## Abstract

Parents transmit genetic and epigenetic information to their offspring. Maternal effect genes regulate the offspring epigenome to ensure normal development. Here we report that the epigenetic regulator SMCHD1 has a maternal effect on *Hox* gene expression and skeletal patterning. Maternal SMCHD1, present in the oocyte and preimplantation embryo, prevents precocious activation of *Hox* genes post-implantation. Without maternal SMCHD1, highly penetrant posterior homeotic transformations occur in the embryo. *Hox* genes are decorated with Polycomb marks H2AK119ub and H3K27me3 from the oocyte throughout early embryonic development; however, loss of maternal SMCHD1 does not deplete these marks. Therefore, we propose maternal SMCHD1 acts downstream of Polycomb marks to establish a chromatin state necessary for persistent epigenetic silencing and appropriate *Hox* gene expression later in the developing embryo. This is a striking role for maternal SMCHD1 in long-lived epigenetic effects impacting offspring phenotype.

## Abstract (web summary)

Parents transmit both genetic and epigenetic information to their offspring, with maternal effect genes being critical regulators of the offspring epigenome. Here they show that maternally deposited SMCHD1 has long-lasting effects on Hox gene expression and vertebral patterning during post-implantation development.

## Introduction

It is now clear that epigenetic information can be passed from generation to generation via the germline, changes in which can have long-lasting effects in the offspring. One of the most notable of these effects is the transmission of epigenetic information from the oocyte to the zygote. The oocyte supplies the entire cytoplasm containing all expressed mRNA and proteins to the zygote, sustaining it through its initial cell divisions until its own zygotic genome is transcribed, at embryonic day (E) 2.0 in mice^1^. Genes whose expression is required in the oocyte for normal development of the offspring are known as maternal effect genes.

A classic example of the role of maternal effect genes in passing long-lived epigenetic information from parent to offspring is genomic imprinting, where genes are monoallelically expressed in a parent-of-origin-specific manner. Epigenetic imprints are imparted by germ cell-derived DNA methylation or trimethylation of lysine 7 on histone 3 (H3K27me3)^2–4^. Maternal effect genes important for imprinting generally have a role in establishing and maintaining these germline marks^5,6^.

*Structural maintenance of chromosomes hinge domain containing 1* (*Smchd1*) is a recently defined maternal effect gene that is expressed in the oocyte and is required for genomic imprinting in the mouse placenta^7–9^. In its zygotic form, SMCHD1 plays a key role in the epigenetic silencing of imprinted loci, along with other clustered gene families and the inactive X chromosome^10–15^. Heterozygous variants in *SMCHD1* are also associated with the human diseases Facioscapulohumeral muscular dystrophy (FSHD) and Bosma arhinia microphthalmia (BAMS)^16–19^, demonstrating the important role SMCHD1 plays in normal development.

SMCHD1 is a member of the SMC family of proteins, large chromosomal ATPases important for chromosome structure^20^. SMCHD1 also plays a role in chromatin architecture, mediating long-range interactions at its targets^12,21–23^. Recruitment to at least one of its targets, the inactive X chromosome, is dependent on the polycomb repressive complex 1 (PRC1) mark ubiquitination of lysine 119 of histone H2A (H2AK119ub)^22,24^. For imprinted genes we have proposed that SMCHD1 is recruited downstream of PRC2’s mark H3K27me3^7^. Precisely how zygotic or maternal SMCHD1 enables gene silencing is not yet clear.

One of the clustered gene families zygotic SMCHD1 binds and silences is the *Hox* genes^11,12,25^, a highly conserved set of transcription factors that are responsible for correct patterning of body segments along the anterior-posterior (A-P) axis during embryonic development^26–29^. *Hox* genes are only expressed at specific times and in specific tissues during post-implantation embryonic development^30,31^. At all other times they are silent and marked by H2AK119ub and H3K27me3^32,33^, including in the oocyte and through pre-implantation development^34–36^, opening the exciting possibility of maternal effects on *Hox* gene expression. Based on these data and *Smchd1*’s role as a maternal effect gene, we investigated whether maternal SMCHD1 has long-lasting effects at its targets in the embryo, specifically on the *Hox* genes. This was made possible because, unlike many maternal effect genes, deletion of maternal *Smchd1* does not result in embryonic lethality^7^.

In this study we show that maternal SMCHD1, found in the preimplantation embryo, is required to prevent premature *Hox* gene activation in the early post-implantation embryo. Interestingly, these changes occurred without disruption of H2AK119ub or H3K27me3 marks over *Hox* genes in the pluripotent state, and without significant loss of these Polycomb marks during differentiation, suggesting that maternal SMCHD1 acts downstream of Polycomb to regulate *Hox* gene expression and normal skeletal patterning post-implantation.

## Results

### Maternal SMCHD1 is required for normal skeletal patterning

**Fig. 1:**
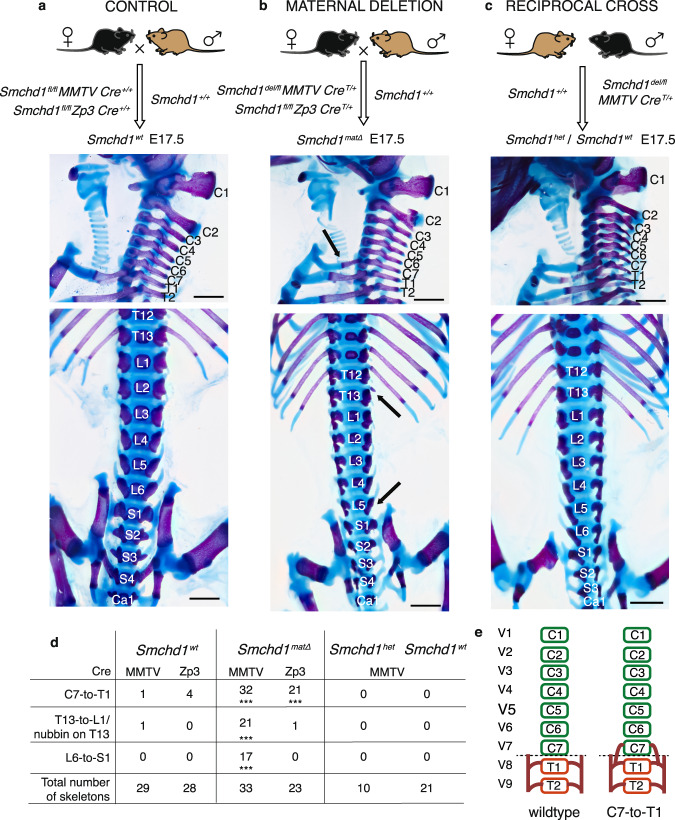
Maternal SMCHD1 is required for normal skeletal patterning. **a**–**c** Upper panels: the genetic crosses used to create control (**a**), maternal null (**b**) and reciprocal cross heterozygous control (**c**) embryos for skeletal analysis. The black mouse represents C57BL/6 strain, the brown mouse Castaneus strain. Middle and lower panels: E17.5 skeletons stained with alizarin red (bone) and alcian blue (cartilage), showing a sagittal view of the cervicothoracic region and dorsal view of the thoraco-lumbar-sacral region. The asterisks in **b** indicate abnormalities compared with the standard axial formulae found in the controls. The skeletons shown are from MMTV-Cre crosses. Scale bar is 5 mm. **d** The summarised data for all skeletons are provided for MMTV and Zp3-Cre. The asterisks indicate statistical significance in comparing to control (two-tailed chi-square test, degrees of freedom = 1, *** *p* < 0.001, *n* = 29 and 28 for the MMTV-Cre and Zp3-Cre control crosses, respectively, *n* = 33 and 23 for the MMTV-Cre and Zp3-Cre maternal deletion crosses respectively, *n* = 10 and 21 for *Smchd1*^*wt*^ and *Smchd1*^*het*^ skeletons respectively from the reciprocal cross using MMTV-Cre). Source data are provided in Supplementary Data 1. **e** Cartoon depiction of normal skeletal patterning and the *Smchd1*^*matΔ*^ phenotype are shown in **e** for the cervico-thoracic region.

Given that previous work in our lab has shown that *Smchd1* mutants exhibit homeotic transformations^12,25^, we first assessed whether *Smchd1* maternal knockout embryos also show abnormal skeletal patterning. We set up F1 crosses between C57BL/6 and Castaneus strain (Cast) parents, using MMTV-Cre or Zp3-Cre to knock out *Smchd1* in the oocyte (Fig. 1a–c) as we have previously^7^. We set up three types of F1 crosses. The first was a control cross yielding embryos with wild-type SMCHD1 function (*Smchd1*^*wt*^). This established a baseline of skeletal patterning in the F1 embryos (Fig. 1a). Almost all of these embryos had normal skeletal patterning, with 97% and 86% of control mice from the MMTV-Cre and Zp3-Cre colonies respectively having the expected 7 cervical vertebrae, 13 thoracic vertebrae, 6 lumbar vertebrae and 4 sacral vertebrae (Fig. 1d). In the second cross, *Smchd1* was deleted in the oocyte with either MMTV-Cre or Zp3-Cre, yielding *Smchd1* heterozygous embryos which lacked maternal SMCHD1 (*Smchd1*^*matΔ*^, Fig. 1b). We analysed skeletal patterning in embryos derived from both Cre models to ensure any phenotype observed was robust. The third cross, performed with the MMTV-Cre only, was reciprocal to the maternal deletion cross and generated both *Smchd1*^*del/+*^(*Smchd1*^*het*^) and *Smchd1*^*wt*^ embryos, with the oocytes from which they were generated having wild-type levels of SMCHD1 (Fig. 1c). This latter cross tested whether any phenotype observed in the *Smchd1*^*matΔ*^ embryos was due to haploinsufficiency for SMCHD1 after zygotic genome activation rather than lack of maternal SMCHD1, and controlled for the direction of the interstrain cross.

*Smchd1*^*matΔ*^ embryos exhibited a highly penetrant addition of a rib on the seventh cervical element (C7), suggesting that C7 adopts the identity of T1 (Fig. 1b, e). We observed several morphological variations of this additional rib including a short ectopic rib, a rib which fused with T1 with and without subsequent bifurcation before joining the sternum, and a full rib which joined the sternum independently of T1 (Supplementary Data 1). Grouped together, any indication of C7 transformation was observed in the MMTV-Cre and Zp3-Cre models at a penetrance of 97% and 91%, respectively (Fig. 1d). Additional posteriorising transformations were observed in a subset of *Smchd1*^*matΔ*^ embryos when *Smchd1* was deleted with MMTV-Cre. These included (*i*) the loss of ribs on T13 leading to a complete T13-to-L1 transformation or severely hypomorphic rib(s) on T13, and (*ii*) a L6-to-S1 transformation. These phenotypes were observed at a lower penetrance than the additional C7 rib; 63% and 52% for the transformation altering T13 and L6 respectively (Fig. 1d). All three of these phenotypes had significantly higher penetrance following the maternal deletion of *Smchd1* compared to both the control and reciprocal cross (p < 0.001, chi-square test), and there was no sex-specificity in the phenotypes observed (Supplementary Data 1). Of note, when a lumbar transformation was observed, it was almost exclusively coincident with transformations at cervicothoracic and thoracolumbar transitions. This suggests serial homeotic transformation in these embryos, supported further by examples of transformation of vertebra surrounding these transition points where specific morphology can be delineated (e.g.  C5-to-C6 and T1-to-T2; Supplementary Data 1). It is unclear why we observed fewer abnormalities and a lower penetrance of the common C7 transformation in the Zp3-Cre model. Potentially the heterozygosity for *Smchd1* (*Smchd1*^*del/fl*^) in the MMTV-Cre model mothers before Cre activation, or the higher genetic heterogeneity (~5% FVB genome) in the MMTV-Cre animals than the Zp3-Cre animals may play a part^7^. Nonetheless, taken together, deletion of maternal *Smchd1* results in a highly penetrant posterior homeotic transformations that can encompass multiple axial regions, implying a potential global shift in patterning effectors. Given there were no abnormalities observed in the *Smchd1* heterozygous skeletons (Fig. 1c, d), these data support the view that maternal SMCHD1 is required for appropriate axial patterning.

We further examined the *Smchd1*^*matΔ*^ embryonic skeletons and did not observe any striking abnormalities beyond those in the axial skeleton. This is consistent with no major abnormalities being observed in *Smchd1* null skeletons previously^12^. Our previous work showed no significant effect of loss of maternal SMCHD1 on viability at mid-gestation or at weaning^7^. Therefore, we chose to focus our attention here on maternal SMCHD1’s regulation of *Hox* genes and axial patterning.

### Maternal SMCHD1 prevents precocious *Hox* gene activation

We investigated whether there were changes in *Hox* gene expression in *Smchd1*^*matΔ*^ embryos that may explain the homeotic transformation phenotype. We first re-analysed our published RNA-sequencing (RNA-seq) data from control and *Smchd1* maternal null E2.75 morula^7^. *Hox* genes were not readily detectable, and there was no change observed in the maternal null compared with control morulae for the single detectable *Hox* gene (*Hoxb13*, Supplementary Figure 1). Given the challenge with detecting low level expression in low input samples, we went on to examine post-implantation developmental stages. The tissue we chose for RNA-sequencing was tailbud tissue of the E8.0-E8.5 embryo, dissected just anterior to the level of the node. This tissue contains the caudal end of the presomitic mesoderm (PSM) and the region harbouring progenitors of the vertebral column, the neuromesodermal progenitors (NMPs)^37,38^. It is the *Hox* expression signatures within cells prior to somite formation that are known to instruct vertebral morphology later in development^39^.

**Fig. 2:**
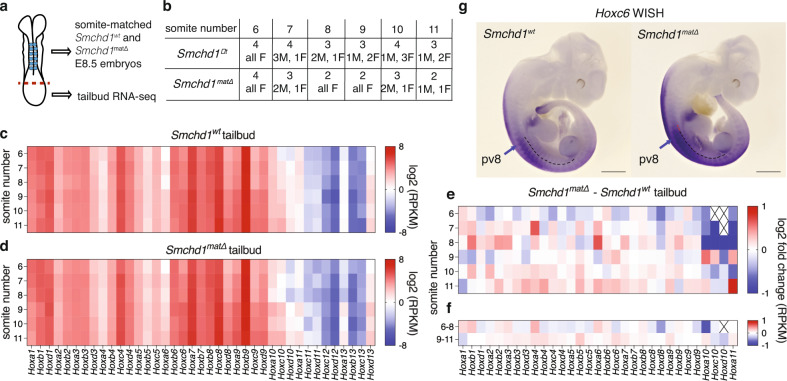
Precocious activation of anterior *Hox* genes in *Smchd1* maternal null tailbud samples. **a** Graphical depiction of an ~E8 embryo, showing somites in blue and dissection point by a dotted red line, and a summary of the experimental approach. **b** Tabular summary of replicate and sex data for each embryo at each somite stage dissected. **c–d** Heatmap of the average reads per million normalised to gene size (RPKM) for the *Hox* genes at 6 to 11 somites in the control (**c**) and *Smchd1* maternally deleted (**d**) tailbud samples from the MMTV-Cre model. The colour scheme is given. **e** Heatmap showing average log2 fold change for expressed *Hox* genes between the *Smchd1* maternal null and control samples. Genes below the expression threshold are denoted with a cross. **f** As in **e** but for grouped somite numbers 6–8, 9–11. Source data are provided in Supplementary Data 2. **g** Whole-mount in situ hybridisation for *Hoxc6* in E10.5 embryos of the given genotypes from the MMTV-Cre model. The arrow indicates prevertebra 8 in each embryo, the red bar marks increased anterior expression of *Hoxc6* in the *Smchd1* maternal null embryo. Scale bar is 1 mm.

We conducted RNA-seq in tailbud tissue from *Smchd1*^*wt*^ and *Smchd1*^*matΔ*^ embryos from the MMTV-Cre model, with somite-matched replicates (Fig. 2a, b, Supplementary Fig. 1). We chose 6–11 somites for two reasons. Firstly, we wanted to focus on our most penetrant and robust change which was observed in the C7 vertebral element. Secondly, we theorised that loss of maternal SMCHD1 may lead to precocious *Hox* gene activation. As we focused on the most anterior and most penetrant change in the absence of maternal SMCHD1, we have used timepoints most appropriate for analysis of anterior *Hox* gene expression. There was very limited differential expression genome-wide in these somite-matched samples. Indeed, what limited differential expression was present can be explained by the sex disparity in samples at each somite number (Supplementary Data 2). Moreover, there was no difference in somite range between control and *Smchd1* maternal null embryos (Supplementary Data 2), suggesting there was no striking developmental delay following loss of maternal SMCHD1. Consistent with this, *Hox* gene expression was approximately normal in *Smchd1*^*wt*^ and *Smchd1*^*matΔ*^ tailbud samples (Fig. 2c, d). As expected, most posterior *Hox* genes are not appreciably expressed at this time (*Hox10*–*13*). When we compared these two somite series, we saw a collective, albeit modest downregulation of *Hox* genes from the *Hox1* to *Hox9* paralogues in 6 somite tissue, followed by the modest upregulation of these genes in somite 8–11 tissue, specifically a trend towards precocious activation of the *Hox2* to *7* paralogues in somite 10 and 11 tissue (Fig. 2e, f). We also observed a concomitant downregulation of posterior *Hox* genes that were detectably expressed (*Hox10* and *11*), particularly at the earlier somite stages (Fig. 2e, f). To confirm and complement these genomic data, we performed whole-mount in situ hybridisation in E10.5 embryos from the MMTV-Cre model for *Hoxc6* (Fig. 2g), one of the upregulated *Hox* genes in the tailbud of 9–11 somite embryos whose expression visually demarcates and functionally patterns the cervico-thoracic transition. Three of four *Smchd1*^*matΔ*^ embryos showed appreciable staining of *Hoxc6* anterior to the normal expression boundary, which was not observed in the four *Smchd1*^*wt*^ controls. These data are consistent with an anterior shift in *Hoxc6* expression boundary in the absence of maternal SMCHD1, the axial skeleton phenotype and tailbud RNA-seq data.

**Fig. 3:**
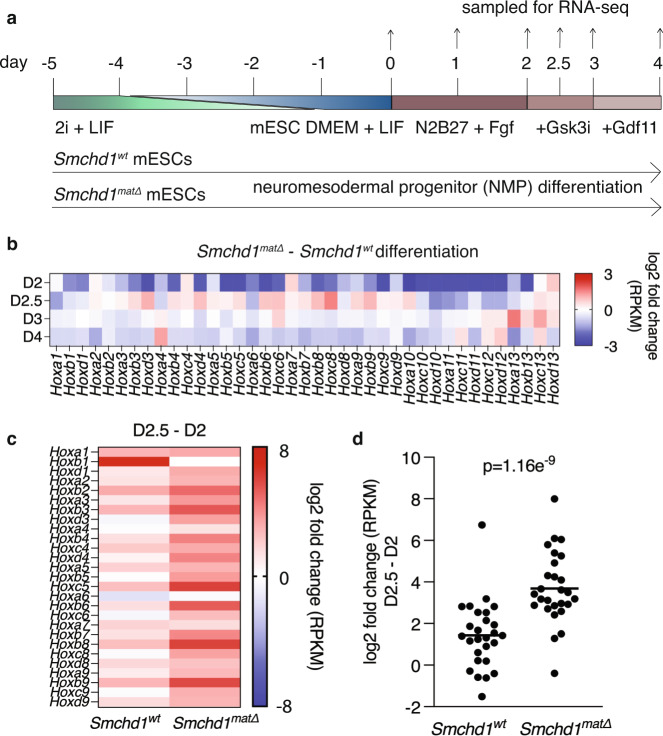
Precocious activation of anterior *Hox* genes in differentiating *Smchd1* maternal null mESCs upon Wnt activation. **a** mESC differentiation to NMP experimental setup, with media components indicated, along with timing of samples taken for RNA-seq. **b** Heatmap of the average log2 fold change for the expressed *Hox* genes between the *Smchd1* maternal null and control samples. *n* = 4 from two technical replicates of two mESC lines derived from separate blastocysts for all days except *Smchd1*^*matΔ*^ day 2.5 where *n* = 3 from technical replicates of two independent mESC lines. **c** Heatmap of the average log2 fold change of *Hox* gene expression between the day 2.5 and day 2 samples, for each genotype. **d** The log2 fold change for the *Hox1*–*9* genes between day 2.5 and day 2 of differentiation (*n* = 27 genes), for the average of the 4 replicates at day 2 and 3 replicates at day 2.5 for each genotype (Student’s t-test, two-tailed, equal variance). Source data are provided in Supplementary Data 3.

To further explore the precocious activation of *Hox* genes in the absence of maternal SMCHD1, we opted for an in vitro approach by differentiating murine embryonic stem cells (mESCs) into neuromesodermal progenitors (NMPs). We derived *Smchd1*^*wt*^ and *Smchd1*^*matΔ*^ mESCs, deleting in the oocyte with Zp3-Cre and performed RNA-seq in the mESCs, where we observed no significantly differentially expressed genes (*n* = 3, Supplementary Data 3, Supplementary Fig. 2). Next, we differentiated the *Smchd1*^*wt*^ and *Smchd1*^*matΔ*^ mESCs, harvesting RNA from differentiating cells every 12 to 24 h from their pluripotent to NMP-like state (*n* = 4, Fig. 3a). Just as observed in vivo, there was no differential expression when analysed genome-wide (Supplementary Fig. 2). The differentiation progressed as expected with the loss of pluripotency factor expression and increase in differentiation factors (Supplementary Fig. 2). At day 2 of differentiation, we observed a subtle decrease in *Hox* gene expression, although these genes were barely expressed above the detection limit of RNA-seq. 12 h after Wnt activation (day 2.5), we observed precocious activation of several anterior *Hox* genes from the *Hox1 to 9* paralogues in the *Smchd1*^*matΔ*^ cells (Fig. 3b, Supplementary Fig. 3), which corresponds to approximately E8.5 in vivo^40^. These combined effects were most noticeable as a larger log fold change between day 2 and day 2.5 of differentiation in the maternal null compared with control cells for anterior *Hox* genes (Fig. 3c, d, *p* < 0.001). At day 3 (NMPs and mesodermal progenitors) and day 4 (24 h after GDF11 addition) corresponding to E9.5 in vivo, we observed a general downregulation of *Hox* gene expression, consistent with the *Hox* gene activation we observe at day 2.5 being precocious but not sustained (Fig. 3b).

To test whether the changes in *Hox* gene expression were due to loss of maternal SMCHD1 or haploinsufficiency for SMCHD1, we generated *Smchd1*^*het*^ and matched control mESC using the MMTV-Cre model with the deleted *Smchd1* allele inherited from the father, as we had for the assessment of axial patterning (Fig. 1a). Using RNA-seq in an identical differentiation series as for the *Smchd1*^*wt*^ and *Smchd1*^*matΔ*^ mESC, we found that *Smchd1*^*het*^ cells differentiated normally and had no significant differential expression when compared with their matched SMCHD1 replete controls (Supplementary Fig. 2). When *Hox* gene expression was examined more specifically, heterozygosity for *Smchd1* did not influence *Hox* gene expression (Supplementary Fig. 3). These data suggest that absence of maternal SMCHD1, rather than haploinsufficiency for SMCHD1, causes precocious *Hox* gene expression during differentiation.

Taken together, these in vivo and in vitro data suggest that the *Smchd1*^*matΔ*^ skeletal phenotype may in part be explained by premature upregulation of anterior *Hox* genes. The normal *Hox* gene silencing in the pluripotent state, and the expected downregulation of *Hox* genes observed later in differentiation, is consistent with the relatively modest effects on skeletal patterning in the absence of maternal SMCHD1.

### Maternal SMCHD1 does not act upstream of Polycomb marks in mESCs

**Fig. 4:**
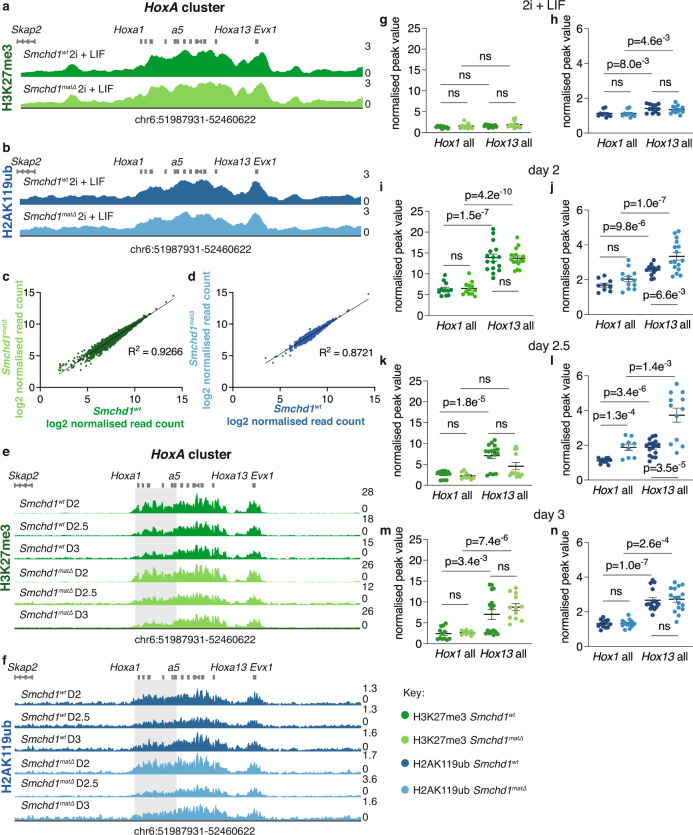
Maternal SMCHD1 does not act upstream of H3K27me3 and H2AK119ub in mESCs. **a** H3K27me3 CUT&RUN in *Smchd1*^*wt*^ and *Smchd1*^*matΔ*^ ESCs cultured in 2i+LIF medium over the *HoxA* cluster as marked. *n* = 3 independent mESC lines per genotype, the average of which is shown. Genes are shown in grey above the CUT&RUN enrichment tracks and genome coordinates are shown below. Green indicates H3K27me3, dark green for *Smchd1*^*wt*^ and light green for *Smchd1*^*matΔ*^. Y-axis is FPM. **b** as in **a** but for H2AK119ub. Blue represents H2AK119ub, dark blue for *Smchd1*^*wt*^ and light blue for *Smchd1*^*matΔ*^. **c** Scatter plot of log2 transformed normalised counts for *Smchd1*^*wt*^ and *Smchd1*^*matΔ*^ over 43,798 H3K27me3 MACS2 peaks in mESCs called from^41^. The Pearson coefficient indicates very high correlation between the two genotypes (*R*^2^ = 0.9266). **d** As in **c ** but for H2AKA119ub, 23,680 peaks called from^42^. Pearson coefficient again indicates very high correlation between the genotypes (*R*^2^ = 0.8721). **e** as in **a** but sampled throughout the mESC to NMP differentiation series at days 2, 2.5 and 3. *n* = 4 from two technical replicates of two mESC lines derived from separate blastocysts for all days except *Smchd1*^*matΔ*^ days 2.5 and 3 where *n* = 3 from technical replicates of two independent mESC lines, the average for which is shown. **f** as in **e** but for H2AK119ub. *n* = 4 from two technical replicates of two mESC lines derived from separate blastocysts for all days except *Smchd1*^*wt*^ day 2 and *Smchd1*^*matΔ*^ day 2.5 where *n* = 3 from technical replicates of two independent mESC lines, the average for which is shown. **g**, **i**, **k**, **m**. Comparison of H3K27me3 CUT&RUN FPKM values over grouped *Hox1* and *Hox13* genes, normalised to FPKM reads over genome-wide MACS peaks within each replicate to correct for efficiency of immunoprecipitation, each for cells grown in 2i + LIF (**i**), harvested at day 2 (**k**), day 2.5 (**m**) and day 3 (**o**). Individual *Hox* genes from each replicate are plotted with mean ± SEM. *P*-values were generated with unpaired, two-tailed t-tests, corrected for multiple testing with the Benjamini-Hochberg method^46^ (ns = not significant, exact *P* values given on graphs for significant results) **h**, **j**, **l**, **n** As in **g**, **i**, **k**, **m** but for H2AK119ub. *n* = 3 per genotype for day 2 and 2.5 and *n* = 4 for day 3 samples, in each case *Hox 1* is 3 genes, *Hox 13* is 4 genes. Source data are provided in Supplementary Data 4, 5 and 6.

To investigate the mechanism by which loss of maternal SMCHD1 caused upregulation of anterior *Hox* genes, we assessed whether the histone marks H2AK119ub and H3K27me3 were perturbed in *Smchd1*^*matΔ*^ mESCs. These histone marks are laid down by Polycomb repressive complex 1 (PRC1) and 2 (PRC2) respectively and induce a heterochromatic gene-silencing state at the *Hox* clusters in mESCs^41,42^. Moreover, both marks are laid down on the maternal chromatin at *Hox* clusters and elsewhere in the genome^34–36^. For non-canonical imprinted genes, maternal H3K27me3 and H2AK119ub are required for their silent state. Based on recent work from our lab on the role of maternal SMCHD1 at non-canonical imprinted genes^7^ we hypothesised that maternal SMCHD1 acts downstream of PRC1 and PRC2. If this were the case, H3K27me3 and H2AK119ub would be unperturbed over *Hox* clusters in *Smchd1*^*matΔ*^ mESCs. We carried out CUT&RUN for H3K27me3 and H2AK119ub in biological triplicate samples of *Smchd1*^*wt*^ and *Smchd1*^*matΔ*^ mESCs, and compared our data to publicly available ChIP-seq datasets for these histone marks in mESCs (Supplementary Data 4^41,42^,). We observed enrichment of H3K27me3 and H2AK119ub at the expected regions for mESCs, including the four *Hox* clusters (Fig. 4a, b, Supplementary Fig. 4); however, we found no change in H3K27me3 or H2AK119ub enrichment between *Smchd1*^*wt*^ and *Smchd1*^*matΔ*^ mESCs over the *Hox* clusters (Fig. 4a, b, Supplementary Fig. 4), and a very high positive correlation between the two genotypes genome-wide (Fig. 4c, d, R^2^ = 0.9266 and 0.8721, respectively). Moreover, there was no significant difference when considering just the maternal or paternal allele of each cluster (Supplementary Fig. 5).

Next, we tested whether loss of maternal SMCHD1 destabilised Polycomb marks during differentiation, which might account for the upregulation of *Hox* genes in its absence. We collected samples for CUT&RUN for H3K27me3 and H2AK119ub at days 2, 2.5 and 3 of mESC differentiation to NMPs, as this is when we observed changes in *Hox* gene expression. It has been shown that H3K27me3 marks are eroded from the anterior end of the *HoxD* cluster as these *Hox* genes are switched on in tailbud tissue^43^, so we expected to see a similar anticorrelation between gene expression and Polycomb coverage in our in vitro differentiation. We observed a subtle decrease in H3K27me3 and H2AK119ub enrichment at the anterior end of each *Hox* cluster during differentiation compared with the pluripotent state (Fig. 4e, f, Supplementary Fig. 6) although with some background signal still present, likely owing to the heterogenous population of cells present in the differentiation culture. To statistically test the differentiation-induced changes in the Polycomb marks and those induced by loss of maternal SMCHD1, we used the csaw package^44^ to call differential peaks both between *Smchd1*^*wt*^ and *Smchd1*^*matΔ*^ samples and between each day of differentiation. This genome-wide method called very few differential peaks between days, none of which were over *Hox* clusters (Supplementary Data 6). Csaw peaks were also called between *Smchd1*^*wt*^ and *Smchd1*^*matΔ*^ samples within each timepoint, again with few significant genome-wide peaks called and none over *Hox* clusters. To look more closely at changes in Polycomb coverage over each *Hox* gene, FPKM values over *Hox* genes (normalised to genome-wide MACS peaks) were compared as a representation of the expected change in anterior to posterior *Hox* gene marking. In the pluripotent mESC we observed no significant difference between the Polycomb mark enrichment at *Hox1* and *Hox13* genes (Fig. 4g, h). At each stage of differentiation, we observed significantly lower enrichment of Polycomb marks at *Hox1* compared with *Hox13* genes, as expected due to activation of the anterior genes during this period (Fig. 4i–n). Interestingly, when comparing each set of *Hox* genes at each timepoint we observed significantly higher coverage of H2AK119ub (but not H3K27me3) at each set of *Hox* genes in *Smchd1*^*matΔ*^ samples compared to *Smchd1*^*wt*^ at days 2 and 2.5 of differentiation (Fig. 4j, l, Supplementary Data 6). This increase in H2AK119ub coverage is not what we would expect given the upregulation of *Hox* genes seen at day 2.5 of differentiation, and is inconsistent with destabilisation of Polycomb marks following loss of maternal SMCHD1, but is suggestive of an interplay between SMCHD1 and Polycomb post-implantation.

We next asked whether alterations to DNA methylation may account for the effects of maternal SMCHD1. First, we re-analysed our previously published whole genome bisulfite sequencing data of E2.75 *Smchd1* maternal null embryos^7,45^ and found very low methylation over all four *Hox* clusters and no difference in methylation between *Smchd1*^*wt*^ and *Smchd1*^*matΔ*^ embryos (Supplementary Fig. 7, Supplementary Data 7). Next, we performed reduced representation bisulfite sequencing at days 2 and 2.5 of differentiation in *Smchd1*^*wt*^ and *Smchd1*^*matΔ*^ differentiating mESCs. Again, we observed very low levels of methylation at the CpG islands at the *Hox* clusters, with no statistically significant difference in DNA methylation between *Smchd1*^*wt*^ and *Smchd1*^*matΔ*^ samples (Supplementary Fig. 7, Supplementary Data 7). These data suggest that maternal SMCHD1 does not influence *Hox* gene expression via modulation of DNA methylation.

Given that the *Smchd1* maternal null mESCs retained their maternal effect on *Hox* gene expression and that loss of maternal SMCHD1 does not disrupt acquisition and/or maintenance of the Polycomb repressive complex marks or CpG island methylation throughout differentiation, within the limitations of our experiments, we conclude that maternal SMCHD1 does not act upstream of Polycomb and DNA methylation in its regulation of *Hox* clusters and other areas of the genome.

## Discussion

In this study we have shown that maternal SMCHD1 is required for appropriate patterning of the axial skeleton, linked to its role in silencing *Hox* genes. Deletion of *Smchd1* in the oocyte results in the highly penetrant posteriorising homeotic transformations of C7-to-T1, T13-to-L1 and L6-to-S1. This phenotype is similar to what is observed in zygotic PRC1 subunit knockouts and is attributable to *Hox* gene overexpression^46,47^. We too observed a modest but consistent upregulation of anterior *Hox* genes both in vivo in the developing tailbud of ~E8.5 embryos and in vitro soon after induction of Wnt signalling in mESC differentiating into NMPs. *Hox* gene silencing was restored later in differentiation, consistent with the relatively subtle axial patterning defects observed in the *Smchd1* maternal null embryos. Interestingly, maternal SMCHD1 was not required to maintain appropriate *Hox* gene silencing earlier in development, either in the pluripotent state or in the morula. These data suggest that maternal SMCHD1, which controls the embryo in the preimplantation period^7,45^, is required to ensure *Hox* genes are not prematurely activated in the post-implantation period. This is a long-lived effect of maternal SMCHD1 at the *Hox* clusters from approximately E2.75 when zygotic SMCHD1 is activated to around E8.5 when precocious *Hox* gene activation is observed.

Given the important role of PRC1 and PRC2 in silencing the *Hox* genes, their role in long-lived mitotic epigenetic memory^48^, and the fact that they mark the *Hox* genes with H2AK119ub and H3K27me3 from the oocyte stage onwards^4,34–36,41^, we asked whether maternal SMCHD1 may function together with the PRCs to silence *Hox* genes, using our mESC model. In undifferentiated mESCs we observed no change in H3K27me3 or H2AK119ub marks genome-wide in *Smchd1* maternally deleted cells compared to control. These data suggest that maternal SMCHD1 acts downstream of Polycomb marks in this system, consistent with previous work showing that zygotic SMCHD1 acts downstream of H2AK119ub on the inactive X chromosome^24^, that H3K27me3 is unchanged over SMCHD1 targets in zygotic *Smchd1* null NSCs^11^, and the role for maternal SMCHD1 at non-canonical imprinted genes controlled by H3K27me3 and H2AK119ub^7^. Potentially, the retention of H2AK119ub and H3K27me3 in the absence of maternal SMCHD1 explains why *Hox* genes are not aberrantly expressed prior to E8.5.

In our embryos we observe a fairly restricted set of posterior homeotic transformations, with the highest penetrance observed for transformations at the anterior end of the skeleton and upregulation of anterior *Hox* genes pertinent to these transformations. Recent studies have reported the dynamic changes in H3K27me3 and H2AK119ub through the preimplantation period, relevant to this consideration^34–36^. Zheng et al. showed that although H3K27me3 decorates the *Hox* clusters in the oocyte and sperm, paternal H3K27me3 is erased and is regained post-implantation, while maternal H3K27me3 remains constantly at *Hox* loci. Mei et al. showed that the same is true for H2AK119ub except it is regained on the paternal allele by the early two-cell stage, faster than paternal H3K27me3 is regained. Considering Polycomb coverage over both alleles, Chen et al. showed both H3K27me3 and H2AK119ub were unchanged from the oocyte to morula stage for the posterior 3’ end of the *HoxC* cluster. Meanwhile, H3K27me3 was erased over the anterior end of the cluster, from the 1-cell to morula stages. H2AK119ub coverage was also reduced, but only at the 1-cell stage. Seeing as this is the window of time when exclusively maternal SMCHD1 protein is present in the embryo^7^, maternal SMCHD1 may affect anterior *Hox* genes because lower levels of H3K27me3 and H2AK119ub create a higher dependence on maternal SMCHD1 for an appropriate chromatin state at the *Hox* genes. However, based on the timepoints sampled in our study, we cannot exclude a role for maternal SMCHD1 in also regulating more posterior *Hox* genes. These studies also show that neither the dynamic changes in Polycomb marks as the early embryo develops, nor the allele-specificity of them, is captured in mESC, as we also find in our CUT&RUN data from mESCs. Hence, although our *Smchd1* maternal null mESCs appear to retain the maternal effect of SMCHD1 as they exhibit anterior *Hox* upregulation, in the future it will be important to study the effects of maternal SMCHD1 in the preimplantation period to fully elucidate the role of maternal SMCHD1 in regulating the *Hox* genes.

Maternal SMCHD1 acting downstream of Polycomb does not fully answer the question of how deletion of *Smchd1* in the oocyte has such a long-lasting effect on the embryo, days after activation of zygotic SMCHD1. Given that zygotic SMCHD1 has a role in maintaining chromatin architecture, specifically long-range chromatin interactions^12,21,23^, including at *Hox* clusters^12^, it is possible that this long-lasting epigenetic memory exists in the form of a particular chromatin conformation at *Hox* clusters that is put in place early in development by maternal SMCHD1. Without maternal SMCHD1, we propose that the chromatin state of the *Hox* clusters is destabilised, leaving *Hox* genes prone to inappropriate activation over time. While the Polycomb marks remain, zygotic SMCHD1 activation at the late morula stage appears to be insufficient to ensure appropriate *Hox* gene silencing later in development. Potentially this is because zygotic SMCHD1 cannot restore the chromatin architecture required for *Hox* silencing at the late morula stage or afterwards, as the establishment of such a chromatin state needs to occur within the context of the dynamic epigenetic reprogramming that happens earlier in preimplantation development. If the Polycomb marks are sufficient for silencing in the short term, why would a maternal SMCHD1-mediated chromatin state be required to prevent premature *Hox* gene activation post-implantation? The early post-implantation period is another time of wholesale epigenome remodelling as the embryo undergoes germ-layer specification and gastrulation. Potentially a destabilised chromatin state created by the absence of maternal SMCHD1 is liable to disruption in the context of such genome-wide remodelling. Our data using CUT&RUN for H3K27me3 and H2AK119ub and reduced representation bisulfite sequencing to analyse DNA methylation at CpG islands during mESC differentiation suggest that these marks are not destabilised in the absence of maternal SMCHD1, at least not to the extent that would allow us to quantitate such a difference in bulk cell populations using our in vitro differentiation system. Therefore, we have not yet been able to identify the specific features of any such destabilised chromatin state. We did observe an enrichment of H2AK119ub, although not H3K27me3, at day 2 and 2.5 of differentiation over *Hox* genes in the absence of maternal SMCHD1. This cannot explain the upregulation of *Hox* genes at this time, but may instead reflect an altered chromatin state, similar to our previously identified enrichment of H3K27me3 on the inactive X chromosome in the absence of zygotic SMCHD1 concomitant with major changes to the architecture of this chromosome^12^. Since we have not examined the Polycomb marks in *Smchd1* heterozygous samples, it remains possible that the subtle changes in H2AK119ub are due to haploinsufficiency for SMCHD1 rather than maternal effects, unlike those on *Hox* gene expression.

Xue et al. recently reported skeletal abnormalities and overexpressed *Hox* genes in the oocyte of a zebrafish model of maternal *Smchd1* knockout^49^. They also showed evidence that zygotic knockout of *Lrif1*, a known binding partner of SMCHD1^50^, phenocopies the *Smchd1* maternal knockout phenotype, suggesting that LRIF1 could be involved in the same regulatory pathway of *Hox* expression as SMCHD1. These complementary results indicate a striking evolutionary conservation of *Hox* genes by maternal SMCHD1 in both mouse and zebrafish, although the mechanism of regulation in each model organism remains unknown.

Although further work is required to elucidate how maternal SMCHD1 has a long-lasting epigenetic memory in the developing embryo, this study shows that maternal SMCHD1 is required for appropriate *Hox* gene expression and, consequently, is also required for normal skeletal patterning in the mouse embryo. This work is relevant to our understanding of how maternal proteins influence offspring phenotypes, and may be relevant to humans considering the pathogenic variants in SMCHD1 observed in several human diseases^16–19^.

## Methods

### Mouse strains and genotyping

Mice were bred, housed and maintained in accordance with standard animal husbandry procedures and experiments performed were approved by the WEHI Animal Ethics Committee under the animal ethics numbers 2018.004, 2020.048 and 2020.50. Mice experienced temperature in the range of 20–22 °C, 30–45% humidity and a 14 h light/10 h dark cycle. *Smchd1*^*del/fl*^ mice carrying the *MMTV-Cre* transgene^51^, and *Smchd1*^*fl/fl*^ mice carrying the *Zp3 Cre* transgene^52^ were created as previously described^7^ and maintained on the C57BL/6 background.

In this study three crosses were carried out to assess the effect of deleting *Smchd1* in the oocyte on the developing embryo. The control cross involved *Smchd1*^*fl/fl*^ *MMTV*-*Cre*^*+/+*^ or *Zp3*-*Cre*^*T/+*^ females and Castaneus (Cast) *Smchd1*^*+/+*^ male mice to generate *Smchd1*^*fl/+*^ (*Smchd1*^*wt*^) embryos. The maternal deletion test cross was carried out with *Smchd1*^*del/fl*^ *MMTV-Cre*^*T/+*^ or *Smchd1*^*fl/fl*^ *Zp3*-*Cre*^*T/+*^ females and Cast *Smchd1*^+/+^ male mice, to generate *Smchd1*^*del/+*^ (*Smchd1*^*matΔ*^) embryos which developed from *Smchd1* homozygous-null oocytes. The third cross was a reciprocal cross between a Cast *Smchd1*^*+/+*^ female and a *Smchd1*^*del/fl*^*; MMTV-Cre*^*T/+*^male to generate *Smchd1*^*fl/+*^ and *Smchd1*^*del/+*^ (*Smchd1*^*het*^) embryos. The reciprocal cross was only conducted with MMTV-Cre, and not Zp3-Cre, with the purpose of controlling for the heterozygosity of *Smchd1*^*matΔ*^ embryos. The F1 nature of the embryos allows for allele-specific genomic analysis due to differential SNPs between the C57BL/6 and Cast genomes.

Genotypes were determined by PCR using GoTaq Green (Promega) for *Smchd1*, the X and Y chromosomes via *Otc* and *Zfy* respectively^12^, and for the *Cre* transgene^53^. TCAGGTGGTCTCGAGCCC, CCATGAGAAGCAATGTGGGA and GGACAGCCAAAGTGACACAG were the oligonucleotides used to detect the *Smchd1* deleted, *Smchd1* floxed and *Smchd1* wild-type alleles. CTGACCGTACACCAAAATTTGCCTG and GATAATCGCGAACATCTTCAGGTTC were the oligonucleotides used to detect the Cre transgene. GTTCTTTCGTTTTCCCCTCTC and GGCATTATCTAAGGAGGAGCATC were used to detect *Otc*, GACTAGACATGTCTTAACATCTGTCC and CCTATTGCATGGACAGCAGCTTATG were used to detect *Zfy*.

### Skeletal preparations

Whole-mount skeletal staining was performed on E17.5 embryos as previously described^54^. Skin and organs were removed, embryos dehydrated and remaining tissue dissolved in acetone. After staining, skeletons were cleared in KOH, washed through a glycerol/water series and imaged in 100% glycerol. Images were acquired with a Vision Dynamic BK Lab System at the Monash University Paleontology Lab. Images were taken with a Canon 5d MkII with a 100 mm Macro lens (focus stop 1:3/1:1). Multiple images were taken to extend the focal depth, and stacked in ZereneStacker using the PMax algorithm. Two people independently scored vertebral formulae of each skeleton, blind to genotype and sex.

### Whole-mount in situ hybridisation

Whole-mount in situ hybridisation was performed as previously described^55^ with some modifications. E10.5 embryos from the MMTV-Cre model were dissected in ice-cold DEPC-treated PBS and fixed in 4% paraformaldehyde (PFA), rotating overnight at 4 °C. All steps were performed rocking at room temperature (RT) unless otherwise specified. Embryos were dehydrated by washing through a graded methanol/DEPC-treated PBS with 1% Tween-20 (PBT) series (25%, 50%, 75%, 100%) for 10 min in each solution. Embryos were then stored at −20 °C until in situ hybridisation, at which point all embryos (both genotypes) were processed in parallel to limit inter-experiment variation. To begin in situ hybridisation, embryos were rehydrated by washing through a reversed graded methanol/PBT series for 10 min in each solution, then washed twice for 5 min in PBT. Embryos were then treated with 10 µg/mL proteinase K for 15 min before being washed twice for 5 min each in PBT, then were post-fixed with 4% PFA and 0.2% glutaraldehyde for 20 min and washed twice for 5 min each in PBT. Embryos were subsequently put in pre-warmed hybridisation solution (50% formamide; 5 x SSC (pH 4.5), 1% SDS; 50 µg/mL heparin; 50 µg/mL yeast tRNA (Sigma, R6750)) at 65 °C for 1 h, then 1 ug/mL DIG-labelled riboprobe was added and embryos were incubated at 65 °C, rocking overnight. The following day, embryos were washed in Solution I (50% formamide; 5 x SSC (pH 4.5); 1% SDS) three times for 30 min each, rocking at 65 °C. Embryos were then washed in Solution II (50% formamide; 2 x SSC (pH 4.5); 0.1% Tween-20) three times for 30 min each, rocking at 65 °C, then were washed in TBS with 1% Tween-20 (TBST) three times for 5 min. Embryos were put in blocking solution (TBST with 10% heat-inactivated sheep serum) for 2 h at RT, then blocking solution was removed and embryos were transferred to blocking solution containing 1:2000 anti-DIG antibody (Roche, 11093274910) then were incubated rocking overnight at 4 °C. The next day embryos were washed in TBST three times for 5 min at RT, five times for 1 h at RT, then overnight at 4 °C. The following day, embryos were washed in NTT (100 mM NaCl, 100 mM Tris-HCl (pH 9.5); 1% Tween-20) 3 times for 10 min before colour development in BM purple (Roche, 11442074001), rocking at room temperature, protected from light for approximately 4 h. Colour development was stopped, at the same time across all samples, by washing in PBT three times for 5 min, then embryos were post-fixed in 4% PFA overnight at 4 °C. Embryos were imaged as described above for skeletal preparations. The plasmid for *Hoxc6* riboprobe generation was a kind gift from P. Sharpe.

### Tailbud dissection

Tailbud dissection and somite counting was performed as previously described^56^. In brief, embryos from the MMTV-Cre model were dissected in ice-cold DEPC-treated PBS. Tailbud tissue was horizontally dissected at a distance of 1.5 somites below the last segmented somite to ensure no contaminating somite tissue was included. Tailbud tissue was snap frozen on dry ice and stored at −80 °C for later RNA extraction. The yolk sac was used for genotyping. Somites were counted before fixing each embryo in 4% DEPC-treated paraformaldehyde at 4 °C overnight. Embryos were washed through a graded methanol/PBT (DEPC- treated PBS with 1% Tween-20 (v/v)) series as described above for whole-mount in situ hybridisation, before brief staining in dilute ethidium bromide solution and imaging under a fluorescence dissection microscope to confirm somite counting.

### mESC derivation and culture

mESCs were derived and cultured as previously described^12,57,58^. Females were superovulated with 5 IU folligon (MSD Animal Health Australia) 2 days before mating and 5 IU chorulon (MSD Animal Health Australia) on the day of mating. E3.5 blastocysts were flushed from the uterine horns of these females with M2 medium (Sigma-Aldrich) and were washed twice in 2i + LIF medium [KnockOut DMEM (Life Technologies), 1 x Glutamax (Life Technologies), 1 x MEM Non-Essential Amino Acids (Life Technologies), 1 x N2 Supplement (Life Technologies), 1 x B27 Supplement (Life Technologies), 1 x Beta- mercaptoethanol (Life Technologies), 100 U/mL Penicillin/100 μg/mL Streptomycin (Life Technologies), 10 μg/mL Piperacillin (Sigma-Aldrich), 10 μg/mL Ciprofloxacin (Sigma- Aldrich), 25 μg/mL Fluconazol (Selleckchem), 1000 U/mL ESGRO Leukemia Inhibitory Factor (Merck), 1 μM StemMACS PD0325901 (Miltenyi Biotech), 3 μM StemMACS CHIR99021 (Mitenyi Biotech)] before each blastocyst was plated in an individual well of a non-tissue culture treated 24-well plate by mouth pipetting. Blastocysts were left for 7 days at 37 °C in a humidified atmosphere with 5% (v/v) carbon dioxide and 5% (v/v) oxygen before outgrowths were picked and washed in trypsin-EDTA for 5 min, then washed in mESC wash media [KnockOut DMEM (Life Technologies), 10% KnockOut Serum Replacement (Life Technologies), 100 IU/mL penicillin/100 μg/mL streptomycin (Life Technologies)], then 2i+LIF media. Outgrowths were mechanically disrupted by pipetting in the 2i + LIF media and then were transferred into a 24-well to be cultured as mESC lines. Cell lines were genotyped to check *Smchd1* knockout and only male lines were selected and these lines were grown in non-tissue culture treated plates in suspension in 2i + LIF medium at 37 °C with 5% (v/v) carbon dioxide and 5% (v/v) oxygen, and were passaged using Accutase (Sigma-Aldrich) every other day.

### Differentiation of mESCs into NMPs

Performed as previously described^12^ with some adaptations. mESCs growing in 2i + LIF medium were passaged onto tissue culture plates coated with 0.1% porcine gelatin (Sigma-Aldrich), in 75% 2i + LIF media and 25% mESC DMEM + LIF media [(high-glucose DMEM, 0.085 mM MEM Non-Essential Amino Acids (Life Technologies), 34 mM NaHCO_3_, 0.085 mM 2-mercaptoethanol (Life Technologies), 100 μg/mL streptomycin, 100 IU/mL penicillin, 15% FBS (Life Technologies), 1,000 U/mL ESGRO leukemia inhibitory factor (Merck), 10 μg/mL piperacillin (Sigma-Aldrich), 10 μg/mL ciprofloxacin (Sigma-Aldrich) and 25 μg/mL fluconazole (Selleckchem)]. 24 h later, medium was changed to 50% 2i + LIF medium and 25% mESC DMEM + LIF medium, then 25% 2i + LIF medium and 75% mESC DMEM + LIF medium 48 h later. The following day (day −1 of differentiation) cells were then split using Accutase (Sigma-Aldrich) and were seeded on 6-well plates and on 13 mm circular glass coverslips (Hecht, cat no. 6.071 724) in 12-well plates coated with 0.1% porcine gelatin (Sigma-Aldrich) at densities of 6.25 × 10^4^ cells/cm^2^ for cells to be harvested at days 0 and 1 of differentiation; and 3 × 10^4^ cells/cm^2^ for cells to be harvested on days 2, 2.5, 3 and 4. 24 h later (day 0 of differentiation) cells were washed with PBS and the medium was changed to N2B27 medium [1:1 mix of Advanced DMEM/F12 (Life Technologies) and Neurobasal medium (Life Technologies), 0.5 × N2 supplement (Life Technologies), 0.5 × B27 supplement, 1 × Glutamax (Life Technologies), 40 μg/mL BSA Fraction V (Life Technologies), 1 × 2-mercaptoethanol (Life Technologies), 100 U/mL penicillin/100 μg/mL streptomycin (Life Technologies), 10 μg/mL piperacillin (Sigma-Aldrich), 10 μg/mL ciprofloxacin (Sigma-Aldrich) and 25 μg/mL fluconazole (Selleckchem) supplemented with 10 ng/mL recombinant human basic FGF (Peprotech)]. On day 2 of differentiation, the medium was changed to N2B27 medium with 10 ng/mL recombinant human basic FGF (Peprotech) and 5 μM StemMACS CHIR99021 (Mitenyi Biotech). On day 3 of differentiation, the medium was changed to N2B27 medium with 10 ng/mL recombinant human basic FGF (Peprotech) and 5 μM StemMACS CHIR99021 (Mitenyi Biotech), and 50 ng/mL recombinant GDF11 (Mitenyi Biotech). Cells were harvested for RNA on days 0, 1, 2, 2.5, 3 and 4 by lysing cells in the 6-well plate in RNA lysis buffer (Zymo) and freezing at −80 °C until extraction. Cells grown on coverslips were fixed for immunofluorescence on days 0, 1, 2, 2.5, 3 and 4 as described in the immunofluorescence methods section.

### Immunofluorescence

Immunofluorescence was performed on differentiating mESCs to NMPs as previously described^12^. In brief, cells grown on coverslips were 13 mm circular glass coverslips (Hecht, cat no. 6.071 724) were washed 3 times for 5 min each in PBS before fixation in 4% paraformaldehyde for exactly 10 min. Fixed cells were then stored in 0.02% sodium azide in PBS at 4 °C for up to a week until all samples from differentiation were ready for processing. Cells were then washed 3 times for 5 min each again in PBS before permeabilisation in 0.5% TritonX in PBS for exactly 5 min on ice. Cells were washed again 3 times for 5 min each in PBS then non-specific binding sites were blocked in 1% bovine serum albumin (Sigma-Aldrich, A9418) in PBS for approximately 1 h at room temperature. Primary antibodies against T/Brachyury (Abcam, #ab209665) and Sox2 (ThermoFisher Scientific, cat #14-9811-82) were then added in 1% BSA solution at a dilution of 1:100 and incubated with cells at 4 °C overnight in a humidified chamber. Cells were then washed 3 times for 5 min each in PBS before incubation with secondary goat anti-rabbit 647 (ThermoFisher Scientific, cat #A21244) and goat anti-rat 568 (Invitrogen, cat #A11077) antibodies each at a dilution of 1:500 in a dark humidified chamber for one hour at room temperature, before washing 3 times for 5 min each again in PBS and counterstaining with DAPI 1:10,000 in PBS for 1 min at room temperature. Cells were washed 3 times for 5 min each again in PBS before mounting on Polysine microscope slides (LabServ, cat #LBSP4981) with Vectashield Vibrance Antifade mounting medium (Vector Laboratories, cat #H-1700). Cells were imaged on an LSM 880 (Zeiss) confocal microscope at 40 x magnification and z-stacks were merged and composite images generated using the ImageJ distribution package FIJI^59^.

### CUT&RUN

CUT&RUN was performed as previously described^60^. mESCs grown in 2i +LIF medium or sampled at days 2, 2.5 and 3 during differentiation were taken out of culture and counted using a haemocytometer before washing by centrifuging at 600 g for 5 min and room temperature then resuspending in wash buffer [20 mM HEPES pH 7.5, 150 mM NaCl, 0.5 mM spermidine and 1 x complete protease inhibitor (Roche)] three times. Approximately 200,000 cells were used for each antibody for replicate 1, and 500,000 each for replicates 2 and 3. 10 uL Concanavalin A-coated beads (Bangs Laboratories, #BP531) per sample were washed in binding buffer (20 mM HEPES pH 7.5, 10 mM KCl, 1 mM CaCl_2_, 1 mM MnCl_2_) then were resuspended in the original volume of beads. 10 uL beads per sample were then bound to the cells in 1 mL wash buffer, nutating for 10 min at room temperature. H3K27me3 (Cell Signalling Technologies, C36B11) and H2AK119ub (Cell Signalling Technologies, D27C40) antibodies were added at a concentration of 1:100 in antibody binding buffer (2 mM EDTA in digitonin wash buffer (wash buffer with 0.025% digitonin) and antibody binding was conducted overnight at 4 °C with both H3K27me3, rotating on a nutator. Samples were then washed three times with digitonin wash buffer before resuspending in digitonin wash buffer with pAG-MNase (EpiCypher, 15–1116) at a concentration of 1:20 and nutating at 4 °C for 1 h to allow pAG-MNase and epitope binding. Samples were then washed twice with digitonin wash buffer then once with low-salt rinse buffer (20 mM HEPES pH 7.5, 0.5 mM spermidine, 0.05% digitonin) before resuspending in 200 uL ice-cold incubation buffer (3.5 mM HEPES pH 7.5, 10 mM CaCl_2_, 0.05% digitonin). Samples were then incubated at 0 °C for exactly 30 min to allow MNase cleavage at antibody bound sites, before resuspension in 200 uL STOP buffer (170 mM NaCl, 20 mM EGTA, 0.05% digitonin, 50 ug/mL RNase A, 25 ug/mL glycogen) then incubation at 37 °C for 30 min. The supernatant containing the cleaved chromatin was separated from the ConA beads, DNA was extracted and purified using phenol/chloroform/isoamylalcohol followed by ethanol and glycogen precipitation, purified DNA was resuspended in 0.1% TE buffer (1 mM Tris-HCl pH 8.0, 0.1 mM EDTA). DNA was quantified with a Qubit dsDNA HS Assay Kit and 6 ng or total DNA if less than 6 ng was used as the input for sequencing library preparation.

Libraries were prepared using the NEBNext Ultra II DNA Library Prep Kit for Illumina (New England Biolabs, E7645), with adapters diluted 1:5 from the supplied concentration. Libraries were quantified on a HS D1000 tape on a 4200 Tapestation (Agilent Technologies) before pooling and sequencing on the Illumina NextSeq platform, with 75 bp paired-end reads.

### CUT&RUN analysis

Adapter trimming of Fastq files was performed using TrimGalore! v0.4.4 with Cutadapt v1.15^61^ and QC carried out using FastQC v0.11.8. Reads were mapped to the GRCm38.p6 version of the mouse reference genome using Bowtie2 v2.3.4.1^62^ and Samtools v1.7^63^. For allele-specific analysis, reads were mapped to the GRCm38 mouse genome reference N-masked for Cast SNPs prepared with SNPsplit v0.3.2^64^. Allele specific bam files were then created, again using SNPsplit v0.3.2^62^. Bam files were imported into SeqMonk v1.47.1, v1.47.2 or v1.48.0^65^. H3K27me3 and H2AK119ub MACS peaks were called from publicly available ChIP-sequencing datasets using the SeqMonk peak caller (settings for 300 bp, *p* < 1 × 10 − 5) (GEO accession number GSE73952^42^, GEO accession number GSE161996), using the input sample as a reference. For analysis of mESCs grown in 2i + LIF medium, three biological replicates each for *Smchd1*^*wt*^ and *Smchd1*^*matΔ*^ were merged in Seqmonk. Peak regions identified above were quantitated in our datasets by read count normalised for library size (corrected to largest datastore) and log2 transformed. Peak quantitations were exported from SeqMonk and correlation scatterplots made using GraphPad Prism v9.0.0. CUT&RUN browser tracks were made by quantifying probes over 1000 bp sliding windows normalised for library size, then smoothed over 20 adjacent windows. For samples collected during differentiation, differential peaks were called between *Smchd1*^*wt*^and *Smchd1*^*matΔ*^ libraries at each differentiation timepoint, and between days for each genotype using csaw^44^. Genome browser views were generated in SeqMonk by merging replicate sets (*n* = 3-4 technical replicates of 2 biological replicate mESC lines per track) with probes called over 500 bp sliding windows smoothed over 5 adjacent windows. FPM was calculated for each window, before normalisation against the average of all MACS peaks for that sample, to correct for efficiency of immunoprecipitation. To compare individual *Hox* genes between days, FPKM was calculated over genes ± 10 kb using the feature probe generator pipeline in SeqMonk. FPKM values for each probe were exported and the value of each *Hox* gene was normalised for efficiency of immunoprecipitation to the genome-wide average of MACS called peak heights for each sample. Graphs were generated using GraphPad Prism v9.0.0. *P*-values were generated with unpaired, two-tailed t-tests, corrected with the Benjamini-Hochberg method^66^.

### RNA-sequencing

RNA-sequencing was carried out on tailbud tissue, 2i mESCs and differentiating cells by first extracting RNA using a Quick-RNA Miniprep Kit (Zymo) with DNase I treatment according to the manufacturer’s instructions. 100 ng total RNA (or less if <100 ng was yielded from tailbud tissue) was used to prepare libraries using either a TruSeq RNA Library Prep Kit v2 (Illumina) or a TruSeq Stranded mRNA kit (Illumina) according to the manufacturer’s instructions. Libraries were size-selected for 200–600 bp and primer dimers cleaned up using Ampure XP beads (Beckman Coulter Life Sciences) and were quantified using a D1000 tape on a 4200 Tapestation (Agilent Technologies). Libraries were then pooled and sequenced on the Illumina NextSeq platform, with 75 bp single-end reads, except for 2i mESC RNA-seq libraries which had paired-end sequencing.

### RNA-sequencing analysis

For tailbud, mESC and NMP differentiation RNA-seq, adapter trimming of Fastq files was performed using TrimGalore! v0.4.4 with Cutadapt v1.15^61^ then QC was carried out using FastQC v0.11.8. Reads were mapped to the GRCm38.p6 version of the mouse reference genome using hisat2 v2.0.5^62^ and Samtools v1.7^63^. Bam files were imported into SeqMonk v1.47.1, v1.47.2 or v1.48.0^64^. Libraries were quantified using Seqmonk’s RNA-seq quantitation pipeline, correcting for total library size and transcript length to generate log2 RPKM values. Differences in *Hox* gene expression were quantified by subtracting *Smchd1*^*wt*^ from *Smchd1*^*matΔ*^ log2 RPKM counts. Heatmaps were generated using GraphPad Prism v9.0.0. Differential gene expression analysis was carried out using the inbuilt edgeR analysis package^65,67^ SeqMonk.

For E2.5 male embryo RNA-seq, data was obtained from and analysed as per^7^. Read were trimmed using TrimGalore v0.4.4 and mapped using hisat2 v2.0.5 to an N-masked version the GRCm38 mouse reference genome for Cast SNPs, made with SNPsplit v0.3.2^68^, in paired-end mode and disabling soft-clipping. Gene counts were obtained from bam files in R v3.5.1^69^ with the featureCounts function from the Rsubread package v1.32.1^70,71^ provided with the GRCm38.90 GTF annotation downloaded from Ensembl, ignoring multi-mapping or multi-overlapping reads. Gene counts were normalized in edgeR v3.24.0^65,67^ with the TMM method^72^. Differential gene expression between the *Smchd1* maternally deleted and wildtype embryos was performed using the glmFit and glmLRT functions^73,74^. *P*-values were corrected with the Benjamini-Hochberg method^66^.

### Reduced representation bisulfite sequencing

Cells were harvested at days 2 and 2.5 of differentiation by dissociation with Accutase (Sigma-Aldrich) for 5 min at 37 °C, washing with mESC wash media [KnockOut DMEM (Life Technologies), 10% KnockOut Serum Replacement (Life Technologies), 100 IU/mL penicillin/100 μg/mL streptomycin (Life Technologies)] then pelleting at 500 g for 5 min. Supernatant was removed and the cell pellet was frozen at −80 °C until extraction. DNA was extracted using a Quick-DNA Miniprep kit, cleaned using a Zymo research DNA Clean and concentrator-5 kit then quantified with a Qubit dsDNA assay kit (Thermo Fisher Scientific Q32853). 100 ng DNA was used as the input for library preparation with the NuGEN Ovation RRBS methyl-seq system (Integrated sciences). Bisulfite conversion was carried out using a QIAGEN EpiTect Fast DNA Bisulfite Kit. Libraries were quantified using a D5000 tape on TapeStation 2200 (Agilent Technologies). Samples were sequenced on the Illumina NextSeq platform using 75 bp single-end reads.

### DNA methylation analysis

For Reduced Representation Bisulfite Sequencing (RRBS), adapter trimming of Fastq files was performed using TrimGalore! v0.4.4 with Cutadapt v1.15^61^ then trimming of the diversity bases introduced by library preparation with the NuGEN Ovation RRBS methyl-seq kit was carried out using the trimRRBSdiversityAdaptCustomers.py script provided by NuGEN^75^. Reads were mapped to the GRCm38.p6 version of the mouse reference genome using Bismark v0.20.0^76^ and methylation calls were extracted using Bismark’s bismark_methylation_extractor function. Bismark.cov files were opened in Seqmonk and % methylation was calculated using Seqmonk’s Bisulphite methylation over features pipeline, filtering probes with over 10 reads for all libraries. % methylation files were exported and graphs were generated using GraphPad Prism v9.0.0. *P*-values were corrected with the Benjamini-Hochberg method^66^. Whole Genome Bisulfite Sequencing (WGBS) data from E2.75 male and female *Smchd1*^*wt*^ and *Smchd1*^*matΔ*^ embryos (*n* = 6 *Smchd1*^*wt*^ females, *n* = 5 *Smchd1*^*wt*^ males, *n* = 4 *Smchd1*^*matΔ*^ females, *n* = 8 *Smchd1*^*matΔ*^ males) was obtained from^7,45^ (BioProject accession PRJNA530651 for male data^45^, GEO accession number GSE186315 for female data). Reads from male and female embryos of each genotype were added and Bismark files were generated and analysed for % DNA methylation over the *Hox* clusters as described above for RRBS data. *P*-values were generated with unpaired, two-tailed t-tests, corrected with the Benjamini-Hochberg method^66^.

### Statistics and reproducibility

No statistical method was used to predetermine sample size. No data were excluded from analyses. The experiments were not randomized. Investigators were blinded to allocation during experiments and outcome assessment for the skeletal scoring. All experiments were replicated at least twice but usually at least three times.

### Reporting summary

Further information on research design is available in the Nature Research Reporting Summary linked to this article.

## Supplementary information

Supplementary Information

Description of Additional Supplementary Files

Supplementary Data 1

Supplementary Data 2

Supplementary Data 3

Supplementary Data 4

Supplementary Data 5

Supplementary Data 6

Supplementary Data 7

Reporting Summary

## Data Availability

All genomic data is available on the Gene Expression Omnibus under number GSE183740. Source data associated with each graph are provided in the Supplementary Data.
