# Supplementary material for: Maternal SMCHD1 regulates Hox gene expression and patterning in the mouse embryo
Source: Nat Commun. 2022 Jul 25;13:4295. doi: 10.1038/s41467-022-32057-x (PMC9314430; doi:10.1038/s41467-022-32057-x)
Supplement: Supplementary file 1 — Supplementary Information [file 41467_2022_32057_MOESM1_ESM.pdf]

Maternal SMCHD1 regulates *Hox* gene expression and patterning in the mouse embryo

Natalia Benetti[1,2], Quentin Gouil[1,2], Andres Tapia del Fierro[1,2], Tamara Beck[1], Kelsey Breslin[1], Andrew Keniry[1,2], Edwina McGlinn[3,4*] and Marnie E. Blewitt[1,2*]

1. The Epigenetics and Development Division, WEHI, Parkville VIC, Australia.
2. The Department of Medical Biology, The University of Melbourne, Parkville VIC, Australia.
3. EMBL Australia, Monash University, Clayton, VIC Australia.
4. Australian Regenerative Medicine Institute, Monash University, Clayton, VIC Australia.

* co-corresponding authors, blewitt@wehi.edu.au and edwina.mcglinn@monash.edu

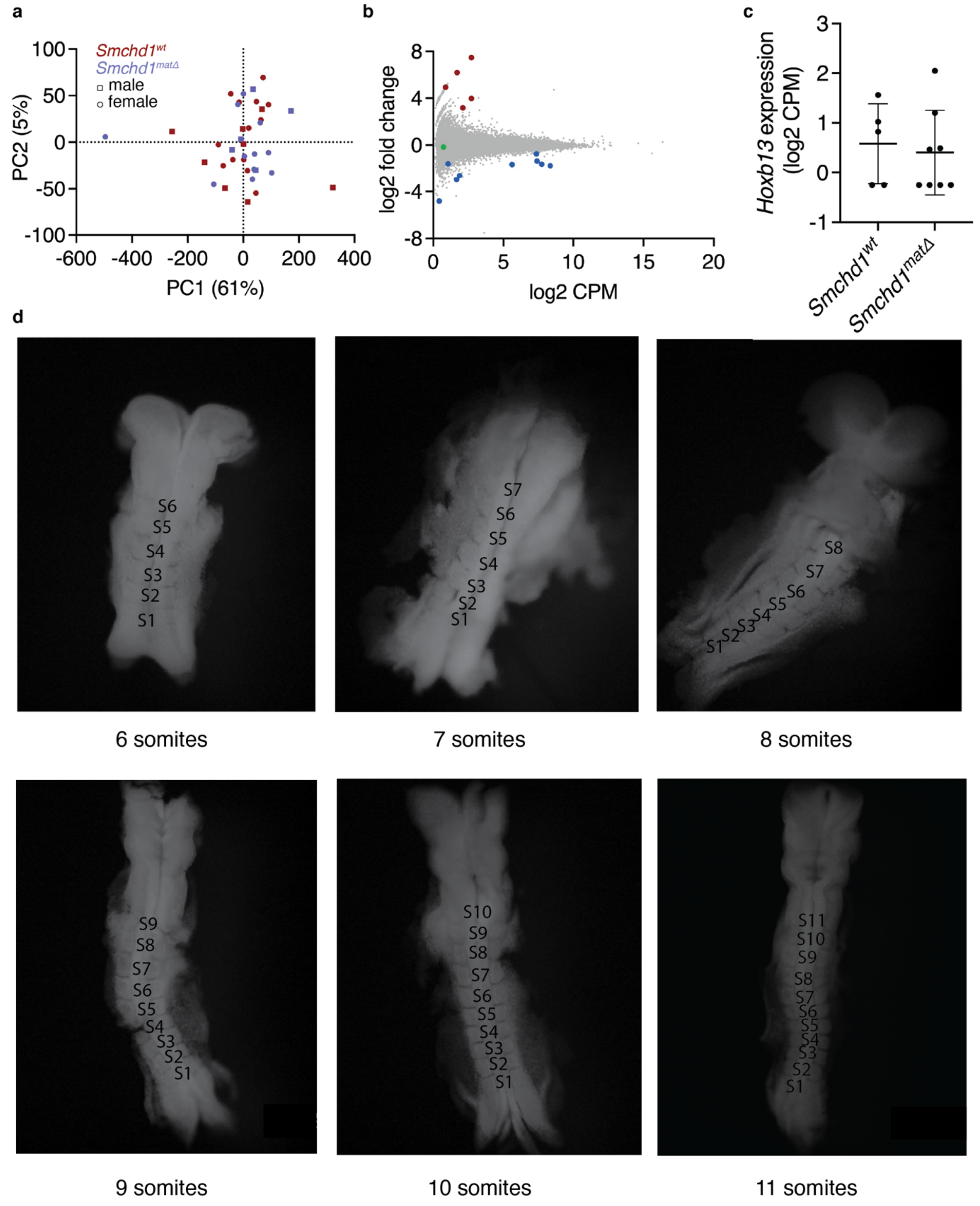

Supplementary Figure 1. RNA-seq expression analysis in embryos. a. Principal Component analysis (PCA) of all RNA-seq samples from E8.0-8.5 tailbud. Red squares represent control $Smchd1^{wt}$ males, red circles represent $Smchd1^{wt}$ females, purple squares represent $Smchd1^{mat\Delta}$ males, purple circles represent $Smchd1^{mat\Delta}$ females. **b**. MA plot of differential expression in $Smchd1^{mat\Delta}$ males compared with $Smchd1^{wt}$ E2.75 embryos, from data published in [7]. The x-axis shows log counts per million (CPM), while the y-axis is the log2 fold change between the $Smchd1^{mat\Delta}$ and control embryos. Significantly

differentially expressed genes are shown in red (upregulated) and blue (downregulated). *Hoxb13* is the only detectable *Hox* gene and is shown in green. Source data are provided in Supplementary Data 1. **c**. *Hoxb13* expression in logCPM in E2.75 male embryos from (b), mean plotted with SD (n=5 and 8 individual embryos for $Smchd1^{wt}$ and $Smchd1^{mat\Delta}$ respectively). **d.** Representative dorsal-view images of E8.0-8.5 embryos at each somite stage after tailbud dissection. Note the ventral view of the 8 somite embryo is shown.

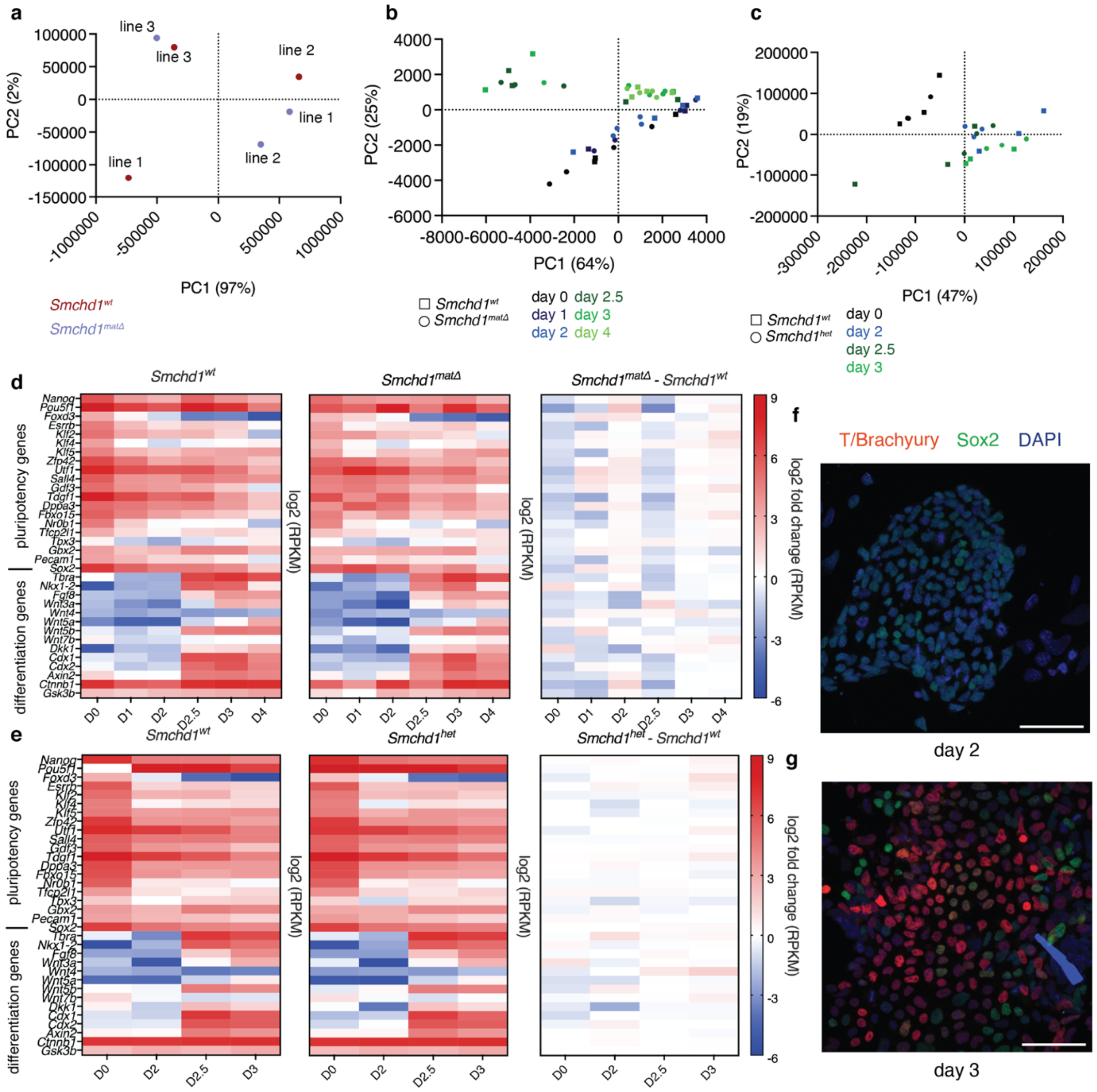

Supplementary Figure 2. Analysis of expression of differentiation factors for differentiating *Smchd1*^{wt}, *Smchd1*$^{mat\Delta}$ and *Smchd1*^{het} male mESC. a. PCA plot for RNA-seq performed on *Smchd1*^{wt} and *Smchd1*$^{mat\Delta}$ male mESC maintained in 2i+LIF medium. n=3 independent mESC lines per genotype. **b**. PCA plot for RNA-seq time course from day 0 to day 4 of differentiation as shown in Figure 3a. n=4 from two technical replicates of two mESC lines derived from separate blastocysts for all days except *Smchd1*^{wt} day 1 and *Smchd1*$^{mat\Delta}$ days 0 and 2.5 where n=3 from technical replicates of two independent mESC lines. **c**. As in (b) but for *Smchd1*^{wt} and *Smchd1*^{het} male mESC generated from the reciprocal of the maternal deletion cross. n=3 independent mESC lines per genotype. **d.** Heatmaps of average RPKM of differentiation and pluripotency factor gene expression, in *Smchd1*^{wt} and *Smchd1*$^{mat\Delta}$ samples, and the average log2 fold change between these two genotypes. Differentiation and pluripotency factor list compiled from [40, 55, 56]. Source data is provided in Supplementary Data 3. **e.** As in (d) but for for

Smchd1^{wt} and *Smchd1*^{het} male mESC generated from the reciprocal of the maternal deletion cross as shown in Figure 1c. Source data are provided in Supplementary Data 4. **f-g**. Immunofluorescence (IF) images of Sox2 and T/Brachyury at days 2 (f) and 3 (g) of differentiation, representative of IF experiments from n=2 of the differentiation experiments from independent mESC lines, each for *Smchd1*^{wt} and *Smchd1*$^{mat\Delta}$. Scale bar is 50 µm.

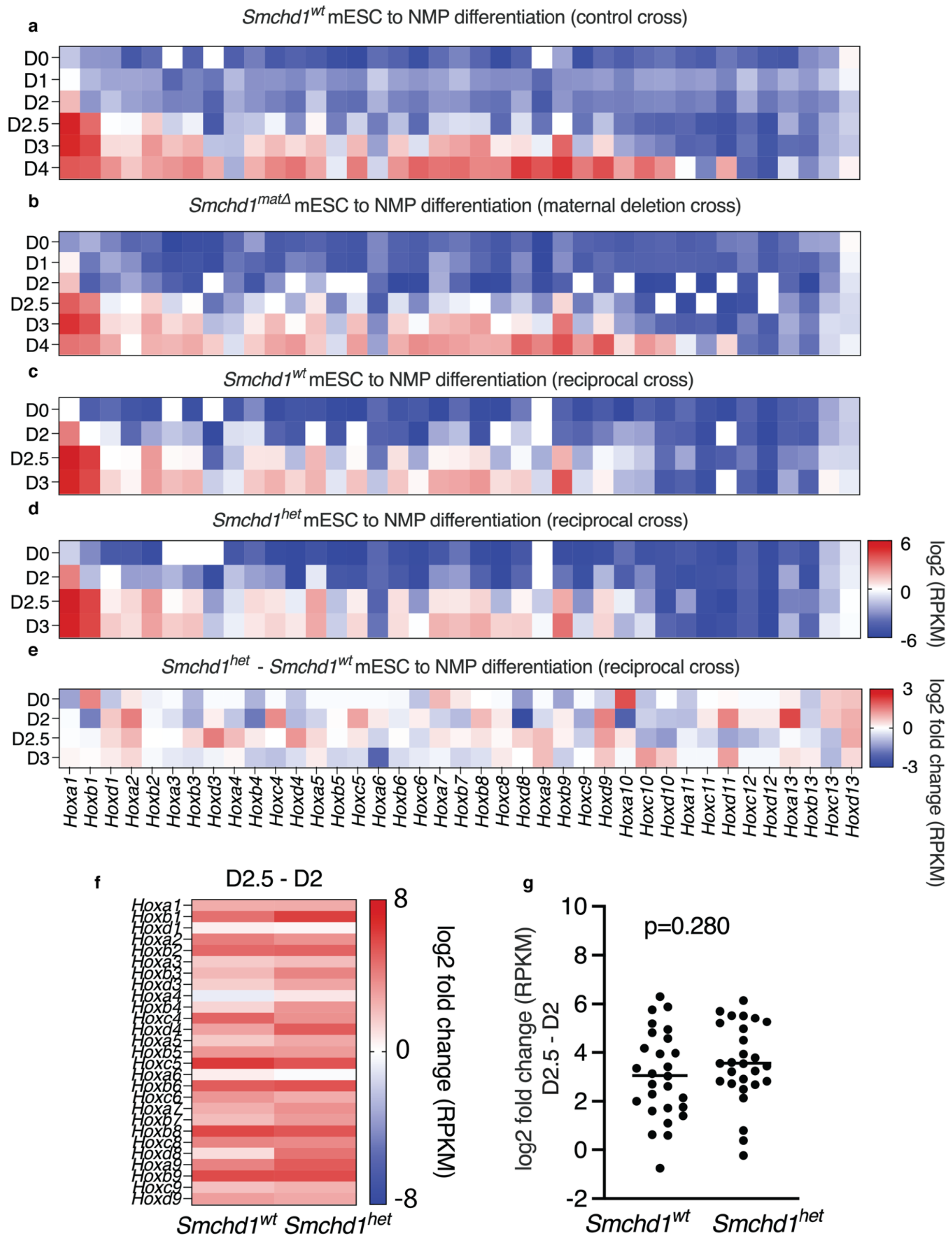

Supplementary Figure 3. Expression analysis for differentiating *Smchd1*^{wt}, *Smchd1*$^{mat\Delta}$ and *Smchd1*^{het} male mESC. a,b. Heatmaps of average log2 RPKM values over all Hox genes during the RNA-seq time course from day 0 to day 4 of differentiation as shown in Figure 3a. n=4 from two

technical replicates of two mESC lines derived from separate blastocysts for all days except *Smchd1*^{wt} day 1 and *Smchd1*$^{mat\Delta}$ days 0 and 2.5 where n=3 from technical replicates of two independent mESC lines, shown for *Smchd1*^{wt} (a) and *Smchd1*$^{mat\Delta}$ (b) mESCs generated from the control and maternal deletion crosses as shown in Figure 1a-b respectively. **c, d.** As in (a) but for *Smchd1*^{wt} (c) and *Smchd1*^{het} (d) mESCs generated from the reciprocal of the maternal deletion cross as shown in Figure 1c. n=3 independent mESC lines per genotype, sampled at days 0, 2, 2.5 and 3 of differentiation. **e.** Heatmaps of the average log2 fold change of Hox genes between *Smchd1*^{wt} (c) and *Smchd1*^{het} samples. **f.** Heatmap of the average log2 fold change of *Hox* gene expression between the day 2.5 and day 2 samples, for *Smchd1*^{wt} and *Smchd1*^{het} samples. **g**. The log2 fold change for the *Hox1-9* genes between day 2.5 and day 2 of differentiation, for the average of the replicates for each genotype (Student's t-test, two-tailed, equal variance). Source data are provided in Supplementary Data 3 and 4.

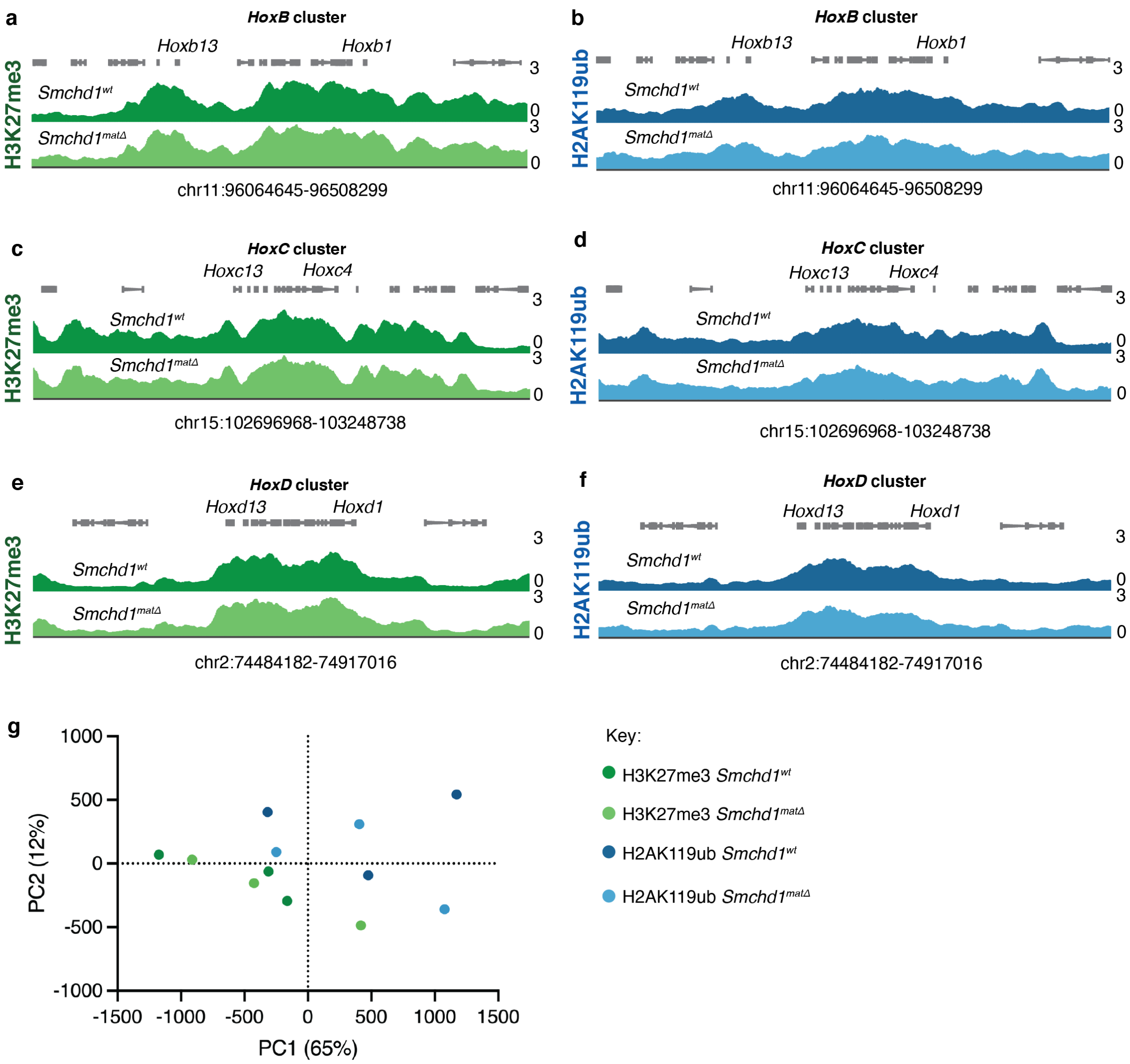

Supplementary Figure 4. H3K27me3 and H2AK119ub coverage over Hox clusters in 2i + LIF mESCs. a, c, e. H3K27me3 CUT&RUN in *Smchd1^{wt}* and *Smchd1$^{mat\Delta}$* ESCs cultured in 2i+LIF medium over the *HoxB* (a), *HoxC* (c) *and HoxD* (e) clusters as marked. n=3 independent mESC lines per genotype, the average of which is shown. Genes are shown in grey above the CUT&RUN enrichment tracks and genome coordinates are shown below. Green indicates H3K27me3, dark green for *Smchd1^{wt}* and light green for *Smchd1$^{mat\Delta}$*. Y-axis is FPM. **b,d,f.** as in (a, c, e) but for H2AK119ub. Blue represents H2AK119ub, dark blue for *Smchd1^{wt}* and light blue for *Smchd1$^{mat\Delta}$*. **g.** PCA plot for H3K27me3 and H2AK119ub CUT&RUN in *Smchd1^{wt}* and *Smchd1$^{mat\Delta}$* mESCs after quantification of 10kb probes sliding by 10kb windows genome-wide, corrected for total read count (FPM). Source data are provided in Supplementary Data 5.

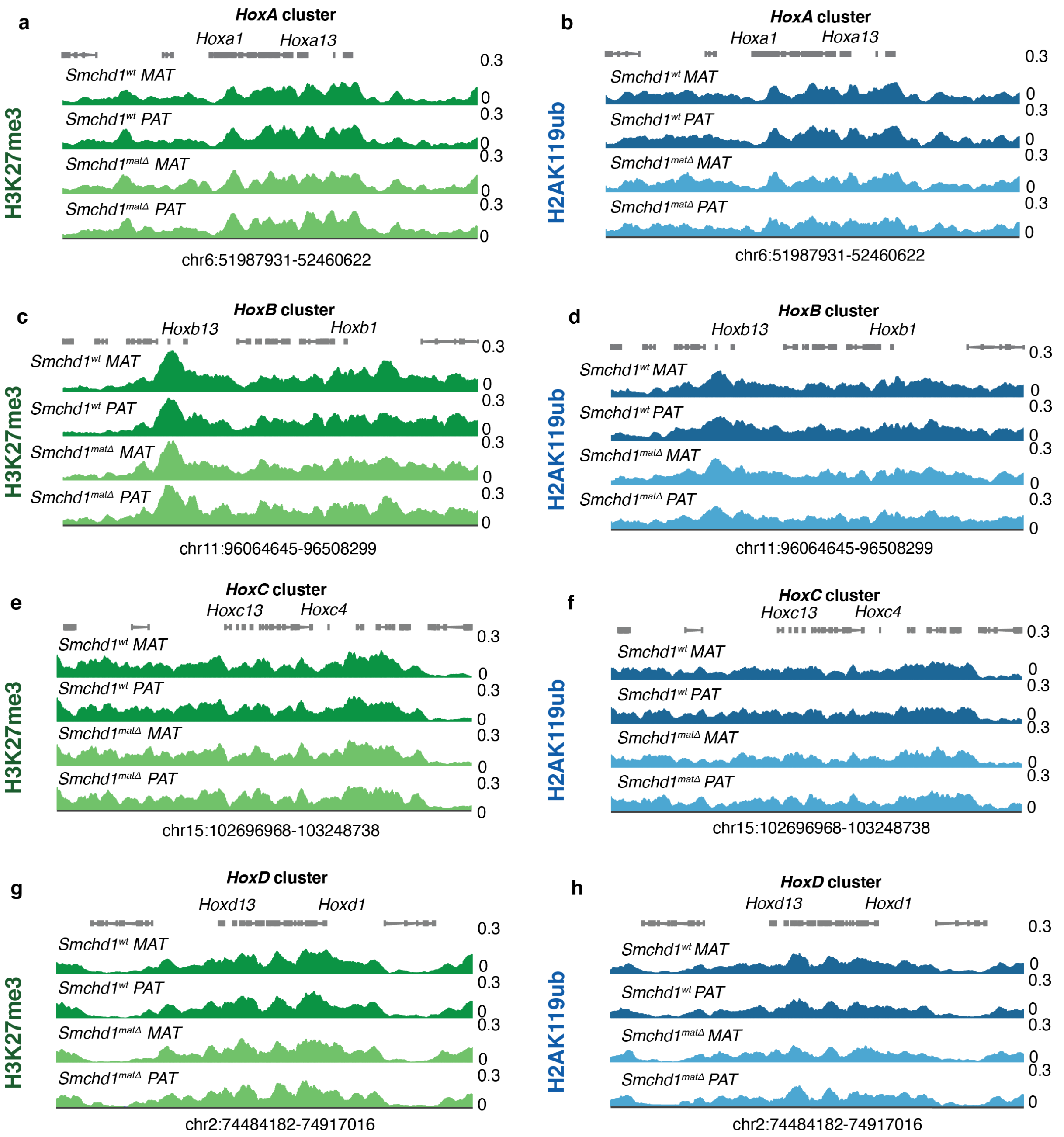

Supplementary Figure 5. H3K27me3 and H2AK119ub over the maternal and paternal allele of each *Hox* cluster. a, c, e, g. CUT&RUN analysis of H3K27me3 over each *Hox* cluster in *Smchd1*^{wt} and *Smchd1*$^{mat\Delta}$ ESCs cultured in 2i+LIF medium, where the reads have been split based on SNPs between the C57BL/6 maternal (MAT) and Cast paternal (PAT) genomes. n=3 independent mESC lines per genotype, the average of which is shown. Genes are shown in grey above the CUT&RUN enrichment tracks, genome coordinates are shown below. Green indicates H3K27me3, dark green for *Smchd1*^{wt} and light green for *Smchd1*$^{mat\Delta}$. Note the read counts are significantly lower than for Figure 4 as reads without an informative SNP are removed. Y-axis is FPM. **b, d, f, h**. as in a. but for H2AK119ub. Blue represents H2AK119ub, dark blue for *Smchd1*^{wt} and light blue for *Smchd1*$^{mat\Delta}$. Source data are provided in Supplementary Data 5.

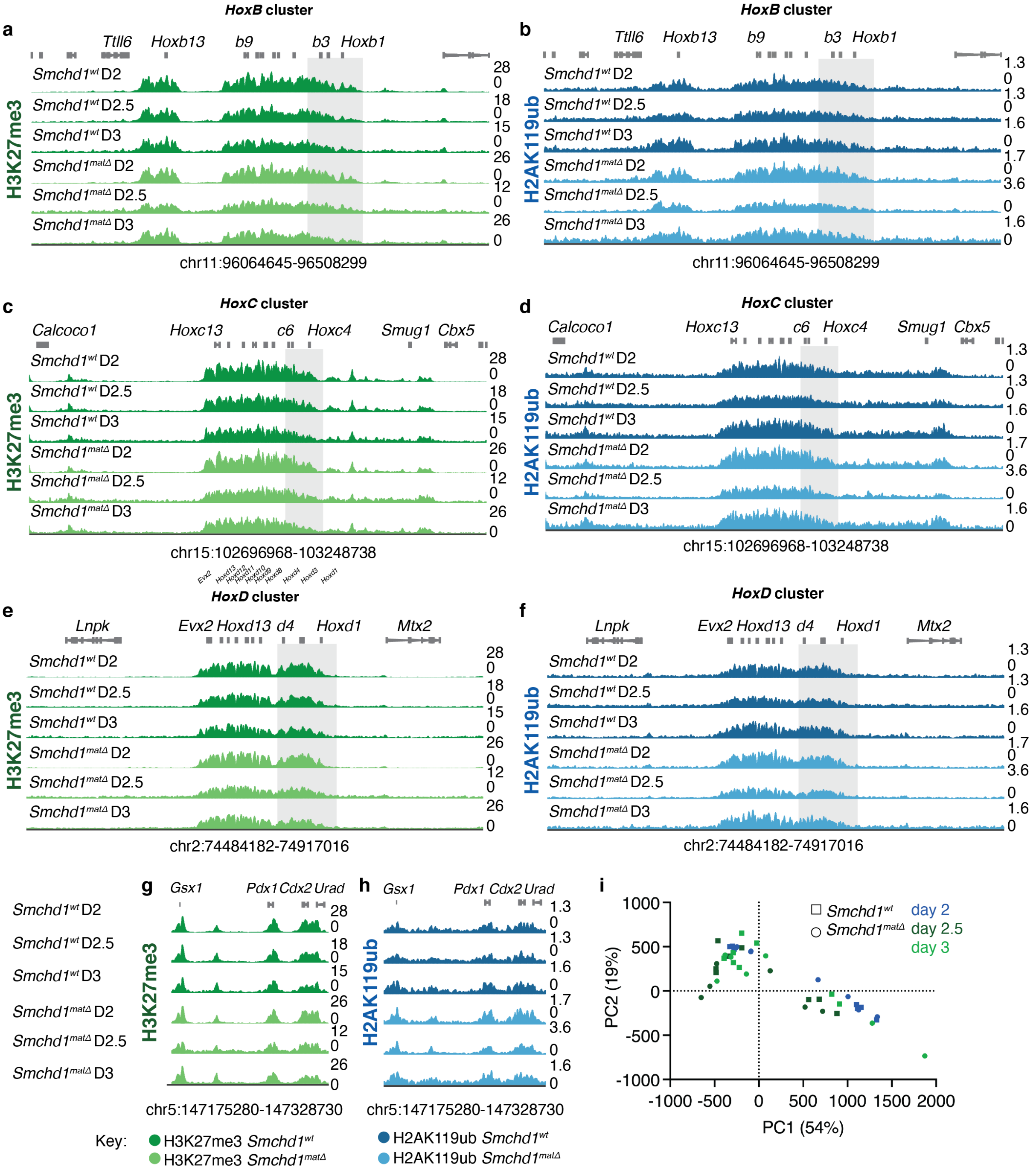

Supplementary Figure 6. H3K27me3 and H2AK119ub coverage over Hox clusters in differentiating mESCs. a, c, e H3K27me3 CUT&RUN in *Smchd1*^{wt} and *Smchd1*$^{mat\Delta}$ ESCs collected at days 2, 2.5 and 3 of differentiation over the *HoxB* (a), *HoxC* (c) *and HoxD* (e) clusters as marked. n=4 from two technical replicates of two mESC lines derived from separate blastocysts for all days except *Smchd1*$^{mat\Delta}$ days 2.5 and 3 where n=3 from technical replicates of two independent mESC lines, the average for which is shown. Genes are shown in grey above the CUT&RUN enrichment tracks and genome coordinates are shown below. Green indicates H3K27me3, dark green for *Smchd1*^{wt} and light green for *Smchd1*$^{mat\Delta}$. **b,d,f.** as in (a, c, e) but for H2AK119ub. n=4 from two technical replicates of two mESC lines derived from separate blastocysts for all days except *Smchd1*^{wt} day 2 and *Smchd1*$^{mat\Delta}$

day 2.5 where n=3 from technical replicates of two independent mESC lines, the average for which is shown. Blue represents H2AK119ub, dark blue for *Smchd1*^{wt} and light blue for *Smchd1*$^{mat\Delta}$. **g.** as in (a) but for genome browser tracks showing *Pdx1* and *Cdx2* for H3K27me3 CUT&RUN as example tracks of a genomic region outside *Hox*. **h.** as in (g) but for H2AK119ub. **i.** PCA plot for H3K27me3 and H2AK119ub CUT&RUN in *Smchd1*^{wt} and *Smchd1*$^{mat\Delta}$ samples collected at days 2, 2.5 and 3 during differentiation, after quantification of 10kb probes sliding by 10kb windows genome-wide, corrected for total read count (FPM). Source data are provided in Supplementary Data 6.

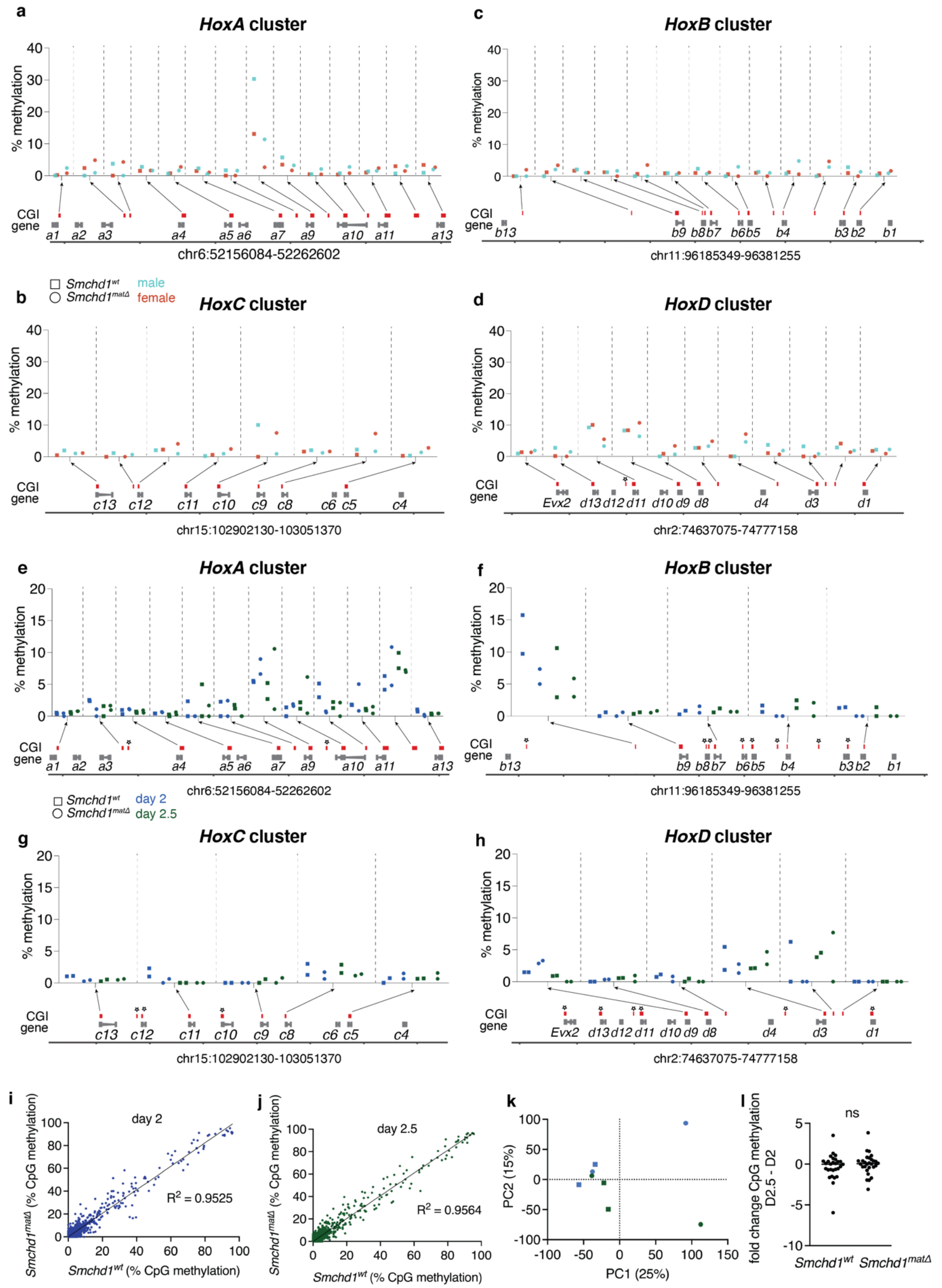

Supplementary Figure 7. DNA methylation of CpG islands is unchanged in *Smchd1* maternal null E2.75 embryos and differentiating mESCs. a-d. % DNA methylation levels from whole genome bisulfite sequencing data of *Smchd1*^{wt} (circles) and *Smchd1*$^{mat\Delta}$ (squares) E2.75 embryos at CpG islands over Hoxa (a), Hoxb (b), Hoxc (c) and Hoxd (d) clusters from [7, 45]. Each datapoint displays the complied

reads of n=6 *Smchd1*^{wt} females, n=5 *Smchd1*^{wt} males, n=4 *Smchd1*$^{mat\Delta}$ females and n=8 *Smchd1*$^{mat\Delta}$ male E2.75 embryos deleted with MMTV-Cre. Male embryos are shown in light blue, females in red. Genomic coordinates, genes in grey and CpG islands in red are shown below the % methylation graphs. Stars indicate no coverage over that CpG island. **e-h.** As in a-d but for reduced representation bisulfite sequencing data of *Smchd1*^{wt} (circles) and *Smchd1*$^{mat\Delta}$ (squares) differentiating mESCs at days 2 (blue) and 2.5 (green) of differentiation. n=2 biological replicates per genotype and timepoint. **i,** Scatterplot showing overall low % methylation over CpG islands genome-wide in *Smchd1*^{wt} and *Smchd1*$^{mat\Delta}$ samples at day 2 (blue) of differentiation. The Pearson coefficient indicates very high correlation between the two genotypes (R^2 = 0.9525). **j.** as in (i) but for day 2.5 (green) of differentiation (R^2 = 0.9564). **k.** PCA plot for *Smchd1*^{wt} (circles) and *Smchd1*$^{mat\Delta}$ (squares) bismark files at day 2 (blue) and 2.5 (green) of differentiation. **l.** Fold change in % DNA methylation at CpG islands surrounding *Hox1-9* genes between day 2 and day 2.5 each for *Smchd1*^{wt} and *Smchd1*$^{mat\Delta}$ samples. Source data are provided in Supplementary Data 7.
