# Supplementary material for: Maternal SMCHD1 regulates Hox gene expression and patterning in the mouse embryo
Source: Nat Commun. 2022 Jul 25;13:4295. doi: 10.1038/s41467-022-32057-x (PMC9314430; doi:10.1038/s41467-022-32057-x)
Supplement: Supplementary file 2 — Description of Additional Supplementary Files [file 41467_2022_32057_MOESM2_ESM.pdf]

Description of Additional Supplementary Files

File Name: Supplementary Data 1
Description: Source data for full skeletal scoring data, including comparison of skeletal phenotype in males and females.

File Name: Supplementary Data 2
Description: Raw read count over all genes**,** Log2 RPKM read counts for *Hox* genes, differential gene expression summary for tailbud samples for merged replicate sets, and replicates separately as Source data.

File Name: Supplementary Data 3
Description: Raw read count over all genes, Log2 RPKM read counts for *Hox* genes and pluripotency/differentiation factors for merged replicate sets, and replicates separately for differentiated cells from the control and maternal deletion crosses as Source data.

File Name: Supplementary Data 4
Description: Raw read count over all genes, Log2 RPKM read counts for *Hox* genes and pluripotency/differentiation factors for merged replicates, and replicates separately for differentiated cells from the reciprocal cross as Source data.

File Name: Supplementary Data 5
Description: Raw read count over all genes, Log2 FPKM reads over MACS2 peaks from published datasets for merged 2i + LIF CUT&RUN replicate sets, and replicates separately as Source data.

File Name: Supplementary Data 6
Description: FPKM reads over all genes, FPKM over MACS2 peaks called genome wide, FPKM reads over all genes normalised to FPKM over MACS2 peaks for merged CUT&RUN differentiation replicate sets, and replicates separately as Source data.

File Name: Supplementary Data 7
Description: % methylation over CpG islands genome-wide and over *Hox* genes for E2.75 embryo and mESC differentiation data. Male and female embryos merged for embryo data, replicate seta and replicates separately for differentiation data as Source data.
