# Supplementary material for: Maternal SMCHD1 regulates Hox gene expression and patterning in the mouse embryo
Source: Nat Commun. 2022 Jul 25;13:4295. doi: 10.1038/s41467-022-32057-x (PMC9314430; doi:10.1038/s41467-022-32057-x)
Supplement: Supplementary file 10 — Reporting Summary [file 41467_2022_32057_MOESM10_ESM.pdf]

nature research

Corresponding author(s): Marnie Blewitt
Edwina McGlinn

Last updated by author(s): 7th July 2022

Reporting Summary

Nature Research wishes to improve the reproducibility of the work that we publish. This form provides structure for consistency and transparency in reporting. For further information on Nature Research policies, see our Editorial Policies and the Editorial Policy Checklist.

Statistics

For all statistical analyses, confirm that the following items are present in the figure legend, table legend, main text, or Methods section.

n/a	Confirmed	
☐	☒	The exact sample size (*n*) for each experimental group/condition, given as a discrete number and unit of measurement
☐	☒	A statement on whether measurements were taken from distinct samples or whether the same sample was measured repeatedly
☐	☒	The statistical test(s) used AND whether they are one- or two-sided<br>*Only common tests should be described solely by name; describe more complex techniques in the Methods section.*
☐	☒	A description of all covariates tested
☐	☒	A description of any assumptions or corrections, such as tests of normality and adjustment for multiple comparisons
☐	☒	A full description of the statistical parameters including central tendency (e.g. means) or other basic estimates (e.g. regression coefficient) AND variation (e.g. standard deviation) or associated estimates of uncertainty (e.g. confidence intervals)
☐	☒	For null hypothesis testing, the test statistic (e.g. *F*, *t*, *r*) with confidence intervals, effect sizes, degrees of freedom and *P* value noted<br>*Give P values as exact values whenever suitable.*
☒	☐	For Bayesian analysis, information on the choice of priors and Markov chain Monte Carlo settings
☒	☐	For hierarchical and complex designs, identification of the appropriate level for tests and full reporting of outcomes
☐	☒	Estimates of effect sizes (e.g. Cohen's *d*, Pearson's *r*), indicating how they were calculated

Our web collection on statistics for biologists contains articles on many of the points above.

Software and code

Policy information about availability of computer code

Data collection	No software was used.
Data analysis	Seqmonk v1.47.1, v1.47.2 or v1.48.0 was used to visualise and perform statistical analysis on all RNAseq and CUT&RUN datasets.<br>SNPsplit_ v0.3.2 was used to process allele specific RNA-seq and CUT&RUN<br>Hisat2 v2.0.5 was used to map RNA-seq<br>Bowtie2 v2.3.4.1 and Samtools v1.7 were used to map the CUT&RUN data<br>csaw v1.24.3 was used to call differential peaks in CUT&RUN data<br>trimRRBSdiversityAdaptCustomers.py script provided by NuGEN was used to trim the diversity bases introduced by library preparation with the NuGEN Ovation RRBS methyl-seq kit<br>Bismark v0.20.0 was used to map WGBS and RRBS reads<br>Cutadapt v1.15 and TrimGalore v0.4.4 were used to trim adapter sequences for CUT&RUN and RNA-seq datasets<br>FastQC v0.11.8 was used for QC of CUT&RUN and RNA-seq datasets<br>Graphpad Prism 9.0.0 was used to perform statistical analyses and graph results<br>The edgeR package inbuilt in Seqmonk was used for all differential expression analyses

For manuscripts utilizing custom algorithms or software that are central to the research but not yet described in published literature, software must be made available to editors and reviewers. We strongly encourage code deposition in a community repository (e.g. GitHub). See the Nature Research guidelines for submitting code & software for further information.

Data

Policy information about availability of data

All manuscripts must include a data availability statement. This statement should provide the following information, where applicable:

- Accession codes, unique identifiers, or web links for publicly available datasets
- A list of figures that have associated raw data
- A description of any restrictions on data availability

All genomics data can be found in the Gene Expression Omnibus, under GSE183740. Raw data is associated with all figures and provided in the supplementary datasets. A data availability statement is included in the manuscript.

Field-specific reporting

Please select the one below that is the best fit for your research. If you are not sure, read the appropriate sections before making your selection.

☒ Life sciences ☐ Behavioural & social sciences ☐ Ecological, evolutionary & environmental sciences

For a reference copy of the document with all sections, see nature.com/documents/nr-reporting-summary-flat.pdf

Life sciences study design

All studies must disclose on these points even when the disclosure is negative.

Sample size	We did not perform any statistical analysis method to determine sample size. Rather, the majority of experiments were performed on n≥3, to ensure reproducibility between replicates while being affordable given the genomic data being produced. Some genomic data is provided as n = 2 because of the difficulty in collecting somite-matched embryos of the necessary genotypes, or the large expense of performing RRBS.
Data exclusions	No data were excluded.
Replication	All experiments were replicated at least twice, with most replicated in excess of three times. No experiments were unreplicated or unable to be reproduced.
Randomization	It was not appropriate to randomise samples in our study design as we wished to test the difference between Smchd1 maternal null and control samples.
Blinding	Blinding was performed for scoring skeletons. Blinding was not performed for genomic experiments as all samples are processed identically and so blinding was deemed unnecessary.

Reporting for specific materials, systems and methods

We require information from authors about some types of materials, experimental systems and methods used in many studies. Here, indicate whether each material, system or method listed is relevant to your study. If you are not sure if a list item applies to your research, read the appropriate section before selecting a response.

Materials & experimental systems

n/a	Involved in the study	
☐	☒	Antibodies
☐	☒	Eukaryotic cell lines
☒	☐	Palaeontology and archaeology
☐	☒	Animals and other organisms
☒	☐	Human research participants
☒	☐	Clinical data
☒	☐	Dual use research of concern

Methods

n/a	Involved in the study	
☐	☒	ChIP-seq
☒	☐	Flow cytometry
☒	☐	MRI-based neuroimaging

Antibodies

Antibodies used	anti-H3K27me3 Cell Signalling technologies, C36B11 cat#9733, lot 14<br>anti-H2AK119ub Cell Signalling Technologies, D27C40 cat# 8240, lot 8<br>anti-Sox2 ThermoFisher Scientific, cat #14-9811-82, lot 2213316 clone BTJCE<br>anti-T/Brachyury Abcam, #ab209665, lot GR326840-6<br>goat anti-rabbit 647 ThermoFisher Scientific cat #A21244<br>goat anti-rat 568 ThermoFisher Scientific cat #A11077<br>anti-DIG Roche, cat# 11093274910, lot 16646821

Validation	All antibodies have validation data available from the supplier websites for the methods we used the antibodies for in our experiments i.e. anti-H3K27me3 and anti-H2AK119ub have ChIPseq data (related to CUT&RUN) provided by Cell Signalling technologies, anti-Sox2 has immunofluorescence data provided by ThermoFisher Scientific, Abcam provides immunofluorescence data for anti-T/Brachyury.<br>We additionally validated that each of these primary antibodies behaved as expected in our controls for the experiment, in terms of providing the IF or CUT&RUN signal expected in the mESC differentiation and in the undifferentiated mESC.

Eukaryotic cell lines

Policy information about cell lines

Cell line source(s)	mESCs derived from E3.5 embryos from C57BL/6 Smchd1fl/fl Zp3-Cre T/+ females x Cast males (maternal deletion)<br>mESCs derived from E3.5 embryos from C57BL/6 Smchd1fl/fl Zp3-Cre +/+ females x Cast males (control)<br>mESCs derived from E3.5 embryos from Cast females x C57BL/6 Smchd1 del/fl MMTV-Cre T/+ males (reciprocal cross)
Authentication	These cell lines were authenticated based on control samples behaving as expected and deletion of exon 4 of C57BL/6 Smchd1 in the maternal deletion samples.
Mycoplasma contamination	All cell lines were derived fresh for this study, often multiple times, and therefore were not at risk of mycoplasma. They were therefore not tested for such.
Commonly misidentified lines<br>(See ICLAC register)	N/A

Animals and other organisms

Policy information about studies involving animals; ARRIVE guidelines recommended for reporting animal research

Laboratory animals	Smchd1 del/fl MMTV-Cre mouse strain which is kept on a C57BL/6 background<br>Smchd1 fl/fl Zp3-Cre mouse strain which is kept on a C57BL/6 background<br>Castaneus mouse strain<br>All samples used in this study were derived from embryos, ranging from E3.5 to E18.5. These embryos were derived from adult male mice (age 8 weeks to 1 year of age) mated with adult females (age 6 weeks to 1 year of age). Both male and female embryos were used in the experiments, with the exception of the mESC lines, where only male cell lines were used due to the issues with X chromosome karyotypic stability in female mESC.
Wild animals	No wild animals were used in this study
Field-collected samples	No field collected samples were used in this study
Ethics oversight	All mice were bred and maintained with standard animal husbandry procedures. At WEHI protocols were approved by the WEHI Animal Ethics Committee under animal ethics numbers AEC 2018.004, 2020.050 and 2020.048.

Note that full information on the approval of the study protocol must also be provided in the manuscript.

ChIP-seq

Data deposition

☒ Confirm that both raw and final processed data have been deposited in a public database such as GEO.

☐ Confirm that you have deposited or provided access to graph files (e.g. BED files) for the called peaks.

Data access links<br>*May remain private before publication.*	All genomics data can be found in the Gene Expression Omnibus, under accession number GSE183740. Please note we have not provided BED files or other graph files, as we did not create such files for our work, and instead viewed our data in Seqmonk, which does not require BED files to be created.
Files in database submission	H3K27me3_CUT&RUN_wt_mESC_line_1_R1.fastq.gz<br>H3K27me3_CUT&RUN_wt_mESC_line_1_R2.fastq.gz<br>H3K27me3_CUT&RUN_wt_mESC_line_2_R1.fastq.gz<br>H3K27me3_CUT&RUN_wt_mESC_line_2_R2.fastq.gz<br>H3K27me3_CUT&RUN_wt_mESC_line_3_R1.fastq.gz<br>H3K27me3_CUT&RUN_wt_mESC_line_3_R2.fastq.gz<br>H3K27me3_CUT&RUN_mat_del_mESC_line_1_R1.fastq.gz<br>H3K27me3_CUT&RUN_mat_del_mESC_line_1_R2.fastq.gz<br>H3K27me3_CUT&RUN_mat_del_mESC_line_2_R1.fastq.gz<br>H3K27me3_CUT&RUN_mat_del_mESC_line_2_R2.fastq.gz<br>H3K27me3_CUT&RUN_mat_del_mESC_line_3_R1.fastq.gz<br>H3K27me3_CUT&RUN_mat_del_mESC_line_3_R2.fastq.gz

April 2020

H2AK119ub_CUT&RUN_wt_mESC_line_1.fastq_R1.gz
H2AK119ub_CUT&RUN_wt_mESC_line_1.fastq_R2.gz
H2AK119ub_CUT&RUN_wt_mESC_line_2.fastq_R1.gz
H2AK119ub_CUT&RUN_wt_mESC_line_2.fastq_R2.gz
H2AK119ub_CUT&RUN_wt_mESC_line_3.fastq_R1.gz
H2AK119ub_CUT&RUN_wt_mESC_line_3.fastq_R2.gz
H2AK119ub_CUT&RUN_mat_del_mESC_line_1_R1.fastq.gz
H2AK119ub_CUT&RUN_mat_del_mESC_line_1_R2.fastq.gz
H2AK119ub_CUT&RUN_mat_del_mESC_line_2_R1.fastq.gz
H2AK119ub_CUT&RUN_mat_del_mESC_line_2_R2.fastq.gz
H2AK119ub_CUT&RUN_mat_del_mESC_line_3_R1.fastq.gz
H2AK119ub_CUT&RUN_mat_del_mESC_line_3_R2.fastq.gz
H3K27me3_CUT&RUN_wt_mESC_day_2_line_1_rep_1_R1.fastq.gz
H3K27me3_CUT&RUN_wt_mESC_day_2_line_1_rep_1_R2.fastq.gz
H3K27me3_CUT&RUN_wt_mESC_day_2_line_1_rep_2_R1.fastq.gz
H3K27me3_CUT&RUN_wt_mESC_day_2_line_1_rep_2_R2.fastq.gz
H3K27me3_CUT&RUN_wt_mESC_day_2_line_2_rep_1_R1.fastq.gz
H3K27me3_CUT&RUN_wt_mESC_day_2_line_2_rep_1_R2.fastq.gz
H3K27me3_CUT&RUN_wt_mESC_day_2_line_2_rep_2_R1.fastq.gz
H3K27me3_CUT&RUN_wt_mESC_day_2_line_2_rep_2_R2.fastq.gz
H3K27me3_CUT&RUN_wt_mESC_day_2.5_line_1_rep_1_R1.fastq.gz
H3K27me3_CUT&RUN_wt_mESC_day_2.5_line_1_rep_1_R2.fastq.gz
H3K27me3_CUT&RUN_wt_mESC_day_2.5_line_1_rep_2_R1.fastq.gz
H3K27me3_CUT&RUN_wt_mESC_day_2.5_line_1_rep_2_R2.fastq.gz
H3K27me3_CUT&RUN_wt_mESC_day_2.5_line_2_rep_1_R1.fastq.gz
H3K27me3_CUT&RUN_wt_mESC_day_2.5_line_2_rep_1_R2.fastq.gz
H3K27me3_CUT&RUN_wt_mESC_day_2.5_line_2_rep_2_R1.fastq.gz
H3K27me3_CUT&RUN_wt_mESC_day_2.5_line_2_rep_2_R2.fastq.gz
H3K27me3_CUT&RUN_wt_mESC_day_3_line_1_rep_1_R1.fastq.gz
H3K27me3_CUT&RUN_wt_mESC_day_3_line_1_rep_1_R2.fastq.gz
H3K27me3_CUT&RUN_wt_mESC_day_3_line_1_rep_2_R1.fastq.gz
H3K27me3_CUT&RUN_wt_mESC_day_3_line_1_rep_2_R2.fastq.gz
H3K27me3_CUT&RUN_wt_mESC_day_3_line_2_rep_1_R1.fastq.gz
H3K27me3_CUT&RUN_wt_mESC_day_3_line_2_rep_1_R2.fastq.gz
H3K27me3_CUT&RUN_wt_mESC_day_3_line_2_rep_2_R1.fastq.gz
H3K27me3_CUT&RUN_wt_mESC_day_3_line_2_rep_2_R2.fastq.gz
H3K27me3_CUT&RUN_mat_del_mESC_day_2_line_1_rep_1_R1.fastq.gz
H3K27me3_CUT&RUN_mat_del_mESC_day_2_line_1_rep_1_R2.fastq.gz
H3K27me3_CUT&RUN_mat_del_mESC_day_2_line_1_rep_2_R1.fastq.gz
H3K27me3_CUT&RUN_mat_del_mESC_day_2_line_1_rep_2_R2.fastq.gz
H3K27me3_CUT&RUN_mat_del_mESC_day_2_line_2_rep_1_R1.fastq.gz
H3K27me3_CUT&RUN_mat_del_mESC_day_2_line_2_rep_1_R2.fastq.gz
H3K27me3_CUT&RUN_mat_del_mESC_day_2_line_2_rep_2_R1.fastq.gz
H3K27me3_CUT&RUN_mat_del_mESC_day_2_line_2_rep_2_R2.fastq.gz
H3K27me3_CUT&RUN_mat_del_mESC_day_2.5_line_1_rep_1_R1.fastq.gz
H3K27me3_CUT&RUN_mat_del_mESC_day_2.5_line_1_rep_1_R2.fastq.gz
H3K27me3_CUT&RUN_mat_del_mESC_day_2.5_line_2_rep_1_R1.fastq.gz
H3K27me3_CUT&RUN_mat_del_mESC_day_2.5_line_2_rep_1_R2.fastq.gz
H3K27me3_CUT&RUN_mat_del_mESC_day_2.5_line_2_rep_2_R1.fastq.gz
H3K27me3_CUT&RUN_mat_del_mESC_day_2.5_line_2_rep_2_R2.fastq.gz
H3K27me3_CUT&RUN_mat_del_mESC_day_3_line_1_rep_1_R1.fastq.gz
H3K27me3_CUT&RUN_mat_del_mESC_day_3_line_1_rep_1_R2.fastq.gz
H3K27me3_CUT&RUN_mat_del_mESC_day_3_line_2_rep_1_R1.fastq.gz
H3K27me3_CUT&RUN_mat_del_mESC_day_3_line_2_rep_1_R2.fastq.gz
H3K27me3_CUT&RUN_mat_del_mESC_day_3_line_2_rep_2_R1.fastq.gz
H3K27me3_CUT&RUN_mat_del_mESC_day_3_line_2_rep_2_R2.fastq.gz
H2AK119ub_CUT&RUN_wt_mESC_day_2_line_1_rep_1_R1.fastq.gz
H2AK119ub_CUT&RUN_wt_mESC_day_2_line_1_rep_1_R2.fastq.gz
H2AK119ub_CUT&RUN_wt_mESC_day_2_line_1_rep_2_R1.fastq.gz
H2AK119ub_CUT&RUN_wt_mESC_day_2_line_1_rep_2_R2.fastq.gz
H2AK119ub_CUT&RUN_wt_mESC_day_2_line_2_rep_1_R1.fastq.gz
H2AK119ub_CUT&RUN_wt_mESC_day_2_line_2_rep_1_R2.fastq.gz
H2AK119ub_CUT&RUN_wt_mESC_day_2.5_line_1_rep_1_R1.fastq.gz
H2AK119ub_CUT&RUN_wt_mESC_day_2.5_line_1_rep_1_R2.fastq.gz
H2AK119ub_CUT&RUN_wt_mESC_day_2.5_line_1_rep_2_R1.fastq.gz
H2AK119ub_CUT&RUN_wt_mESC_day_2.5_line_1_rep_2_R2.fastq.gz
H2AK119ub_CUT&RUN_wt_mESC_day_2.5_line_2_rep_1_R1.fastq.gz
H2AK119ub_CUT&RUN_wt_mESC_day_2.5_line_2_rep_1_R2.fastq.gz
H2AK119ub_CUT&RUN_wt_mESC_day_2.5_line_2_rep_2_R1.fastq.gz
H2AK119ub_CUT&RUN_wt_mESC_day_2.5_line_2_rep_2_R2.fastq.gz
H2AK119ub_CUT&RUN_wt_mESC_day_3_line_1_rep_1_R1.fastq.gz
H2AK119ub_CUT&RUN_wt_mESC_day_3_line_1_rep_1_R2.fastq.gz
H2AK119ub_CUT&RUN_wt_mESC_day_3_line_1_rep_2_R1.fastq.gz
H2AK119ub_CUT&RUN_wt_mESC_day_3_line_1_rep_2_R2.fastq.gz
H2AK119ub_CUT&RUN_wt_mESC_day_3_line_2_rep_1_R1.fastq.gz
H2AK119ub_CUT&RUN_wt_mESC_day_3_line_2_rep_1_R2.fastq.gz

H2AK119ub_CUT&RUN_wt_mESC_day_3_line_2_rep_2_R1.fastq.gz
H2AK119ub_CUT&RUN_wt_mESC_day_3_line_2_rep_2_R2.fastq.gz
H2AK119ub_CUT&RUN_mat_del_mESC_day_2_line_1_rep_1_R1.fastq.gz
H2AK119ub_CUT&RUN_mat_del_mESC_day_2_line_1_rep_1_R2.fastq.gz
H2AK119ub_CUT&RUN_mat_del_mESC_day_2_line_1_rep_2_R1.fastq.gz
H2AK119ub_CUT&RUN_mat_del_mESC_day_2_line_1_rep_2_R2.fastq.gz
H2AK119ub_CUT&RUN_mat_del_mESC_day_2_line_2_rep_1_R1.fastq.gz
H2AK119ub_CUT&RUN_mat_del_mESC_day_2_line_2_rep_1_R2.fastq.gz
H2AK119ub_CUT&RUN_mat_del_mESC_day_2_line_2_rep_2_R1.fastq.gz
H2AK119ub_CUT&RUN_mat_del_mESC_day_2_line_2_rep_2_R2.fastq.gz
H2AK119ub_CUT&RUN_mat_del_mESC_day_2.5_line_1_rep_1_R1.fastq.gz
H2AK119ub_CUT&RUN_mat_del_mESC_day_2.5_line_1_rep_1_R2.fastq.gz
H2AK119ub_CUT&RUN_mat_del_mESC_day_2.5_line_2_rep_1_R1.fastq.gz
H2AK119ub_CUT&RUN_mat_del_mESC_day_2.5_line_2_rep_1_R2.fastq.gz
H2AK119ub_CUT&RUN_mat_del_mESC_day_2.5_line_2_rep_2_R1.fastq.gz
H2AK119ub_CUT&RUN_mat_del_mESC_day_2.5_line_2_rep_2_R2.fastq.gz
H2AK119ub_CUT&RUN_mat_del_mESC_day_3_line_1_rep_1_R1.fastq.gz
H2AK119ub_CUT&RUN_mat_del_mESC_day_3_line_1_rep_1_R2.fastq.gz
H2AK119ub_CUT&RUN_mat_del_mESC_day_3_line_1_rep_2_R1.fastq.gz
H2AK119ub_CUT&RUN_mat_del_mESC_day_3_line_1_rep_2_R2.fastq.gz
H2AK119ub_CUT&RUN_mat_del_mESC_day_3_line_2_rep_1_R1.fastq.gz
H2AK119ub_CUT&RUN_mat_del_mESC_day_3_line_2_rep_1_R2.fastq.gz
H2AK119ub_CUT&RUN_mat_del_mESC_day_3_line_2_rep_2_R1.fastq.gz
H2AK119ub_CUT&RUN_mat_del_mESC_day_3_line_2_rep_2_R2.fastq.gz
no_Ab_CUT&RUN_wt_mESC_day_2_line_1_R1.fastq.gz
no_Ab_CUT&RUN_wt_mESC_day_2_line_1_R2.fastq.gz
no_Ab_CUT&RUN_wt_mESC_day_2.5_line_1_R1.fastq.gz
no_Ab_CUT&RUN_wt_mESC_day_2.5_line_1_R2.fastq.gz
no_Ab_CUT&RUN_wt_mESC_day_3_line_1_R1.fastq.gz
no_Ab_CUT&RUN_wt_mESC_day_3_line_1_R2.fastq.gz

Genome browser session
(e.g. UCSC)

No longer applicable

Methodology

Replicates

3 replicates (independent mESC lines derived from individual embryos) of each genotype for each experiment, using CUT&RUN.

Sequencing depth

H3K27me3 CUT&RUN wt mESC line 1: 16218934 total, 12024957 unique, 75 bp paried-end reads
H3K27me3 CUT&RUN wt mESC line 2: 21698426 total, 15262734 unique, 75 bp paried-end reads
H3K27me3 CUT&RUN wt mESC line 3: 25527511 total, 18550171 unique, 75 bp paried-end reads
H3K27me3 CUT&RUN mat del mESC line 1: 6277530 total, 4378657 unique, 75 bp paried-end reads
H3K27me3 CUT&RUN mat del mESC line 2: 24588376 total, 17554736 unique, 75 bp paried-end reads
H3K27me3 CUT&RUN mat del mESC line 3: 14566187 total, 10155359 unique, 75 bp paried-end reads
H2AK119ub CUT&RUN wt mESC line 1: 17594984 total, 13147293 unique, 75 bp paried-end reads
H2AK119ub CUT&RUN wt mESC line 2: 23407036 total, 16555051 unique, 75 bp paried-end reads
H2AK119ub CUT&RUN wt mESC line 3: 16336891 total, 11899732 unique, 75 bp paried-end reads
H2AK119ub CUT&RUN mat del mESC line 1: 6027488 total, 4311639 unique, 75 bp paried-end reads
H2AK119ub CUT&RUN mat del mESC line 2: 24958476 total, 18333399 unique, 75 bp paried-end reads
H2AK119ub CUT&RUN mat del mESC line 3: 19002652 total, 13455859 unique, 75 bp paried-end reads
H3K27me3 CUT&RUN wt mESC day 2 line 1 rep 1: 13073675 total, 12693231 unique ,75 bp paried-end reads
H3K27me3 CUT&RUN wt mESC day 2 line 1 rep 2: 15618264 total, 15141906 unique ,75 bp paried-end reads
H3K27me3 CUT&RUN wt mESC day 2 line 2 rep 1: 11366752 total, 10972325 unique ,75 bp paried-end reads
H3K27me3 CUT&RUN wt mESC day 2 line 2 rep 2: 13337453 total, 12921324 unique ,75 bp paried-end reads
H3K27me3 CUT&RUN wt mESC day 2.5 line 1 rep 1: 31052192 total, 29928102 unique ,75 bp paried-end reads
H3K27me3 CUT&RUN wt mESC day 2.5 line 1 rep 2: 22600915 total, 21714959 unique ,75 bp paried-end reads
H3K27me3 CUT&RUN wt mESC day 2.5 line 2 rep 1: 11803870 total, 11446212 unique ,75 bp paried-end reads
H3K27me3 CUT&RUN wt mESC day 2.5 line 2 rep 2: 12972741 total, 12579666 unique ,75 bp paried-end reads
H3K27me3 CUT&RUN wt mESC day 3 line 1 rep 1: 7278652 total, 6916175 unique ,75 bp paried-end reads
H3K27me3 CUT&RUN wt mESC day 3 line 1 rep 2: 25637832 total, 24309792 unique ,75 bp paried-end reads
H3K27me3 CUT&RUN wt mESC day 3 line 2 rep 1: 6739445 total, 6484020 unique ,75 bp paried-end reads
H3K27me3 CUT&RUN wt mESC day 3 line 2 rep 2: 6523374 total, 6256568 unique ,75 bp paried-end reads
H3K27me3 CUT&RUN mat del mESC day 2 line 1 rep 1: 12057675 total, 11658565 unique ,75 bp paried-end reads
H3K27me3 CUT&RUN mat del mESC day 2 line 1 rep 2: 10119963 total, 9804220 unique ,75 bp paried-end reads
H3K27me3 CUT&RUN mat del mESC day 2 line 2 rep 1: 6042603 total, 5846822 unique ,75 bp paried-end reads
H3K27me3 CUT&RUN mat del mESC day 2 line 2 rep 2: 6746518 total, 6509715 unique ,75 bp paried-end reads
H3K27me3 CUT&RUN mat del mESC day 2.5 line 1 rep 1: 16305698 total, 15708909 unique ,75 bp paried-end reads
H3K27me3 CUT&RUN mat del mESC day 2.5 line 2 rep 1: 15194173 total, 14724673 unique ,75 bp paried-end reads
H3K27me3 CUT&RUN mat del mESC day 2.5 line 2 rep 2: 11527266 total, 11150324 unique ,75 bp paried-end reads
H3K27me3 CUT&RUN mat del mESC day 3 line 1 rep 1: 6661162 total, 6397380 unique ,75 bp paried-end reads
H3K27me3 CUT&RUN mat del mESC day 3 line 2 rep 1: 6811909 total, 6478125 unique ,75 bp paried-end reads
H3K27me3 CUT&RUN mat del mESC day 3 line 2 rep 2: 7447449 total, 7158488 unique ,75 bp paried-end reads
H2AK119ub CUT&RUN wt mESC day 2 line 1 rep 1: 12583710 total, 12217524 unique ,75 bp paried-end reads
H2AK119ub CUT&RUN wt mESC day 2 line 1 rep 2: 12523478 total, 12141512 unique ,75 bp paried-end reads
H2AK119ub CUT&RUN wt mESC day 2 line 2 rep 1: 10960512 total, 10606487 unique ,75 bp paried-end reads
H2AK119ub CUT&RUN wt mESC day 2.5 line 1 rep 1: 11855363 total, 11480734 unique ,75 bp paried-end reads

H2AK119ub CUT&RUN wt mESC day 2.5 line 1 rep 2: 19775532 total, 18984511 unique ,75 bp paried-end reads
H2AK119ub CUT&RUN wt mESC day 2.5 line 2 rep 1: 10228412 total, 9905194 unique ,75 bp paried-end reads
H2AK119ub CUT&RUN wt mESC day 2.5 line 2 rep 2: 10951211 total, 10587631 unique ,75 bp paried-end reads
H2AK119ub CUT&RUN wt mESC day 3 line 1 rep 1: 9477957 total, 9154759 unique ,75 bp paried-end reads
H2AK119ub CUT&RUN wt mESC day 3 line 1 rep 2: 7389391 total, 7088643 unique ,75 bp paried-end reads
H2AK119ub CUT&RUN wt mESC day 3 line 2 rep 1: 7253488 total, 6993813 unique ,75 bp paried-end reads
H2AK119ub CUT&RUN wt mESC day 3 line 2 rep 2: 6227374 total, 6023116 unique ,75 bp paried-end reads
H2AK119ub CUT&RUN mat del mESC day 2 line 1 rep 1: 10636827 total, 10318786 unique ,75 bp paried-end reads
H2AK119ub CUT&RUN mat del mESC day 2 line 1 rep 2: 8855290 total, 85639501 unique ,75 bp paried-end reads
H2AK119ub CUT&RUN mat del mESC day 2 line 2 rep 1: 6704012 total, 6464008 unique ,75 bp paried-end reads
H2AK119ub CUT&RUN mat del mESC day 2 line 2 rep 2: 4647888 total, 4478240 unique ,75 bp paried-end reads
H2AK119ub CUT&RUN mat del mESC day 2.5 line 1 rep 1: 15562743 total, 14835963 unique ,75 bp paried-end reads
H2AK119ub CUT&RUN mat del mESC day 2.5 line 2 rep 1: 11761070 total, 11160079 unique ,75 bp paried-end reads
H2AK119ub CUT&RUN mat del mESC day 2.5 line 2 rep 2: 15878506 total, 15360867 unique ,75 bp paried-end reads
H2AK119ub CUT&RUN mat del mESC day 3 line 1 rep 1: 5913060 total, 5712016 unique ,75 bp paried-end reads
H2AK119ub CUT&RUN mat del mESC day 3 line 1 rep: 5466485 total, 5284451 unique ,75 bp paried-end reads
H2AK119ub CUT&RUN mat del mESC day 3 line 2 rep 1: 5891187 total, 5679104 unique ,75 bp paried-end reads
H2AK119ub CUT&RUN mat del mESC day 3 line 2 rep 2: 3612792 total, 3485622 unique ,75 bp paried-end reads
no Ab CUT&RUN wt mESC day 2 line 1: 4425796 total, 4281515 unique ,75 bp paried-end reads
no Ab CUT&RUN wt mESC day 2.5 line 1: 12545546 total, 12130288 unique ,75 bp paried-end reads
no Ab CUT&RUN wt mESC day 3 line 1: 7134268 total, 6836056 unique ,75 bp paried-end reads

Antibodies

anti-H3K27me3 Cell Signalling technologies, C36B11 cat#9733
anti-H2AK119ub Cell Signalling Technologies, D27C40 cat# 8240

Peak calling parameters

H3K27me3 and H2AK119ub MACS peaks were called from publicly available ChIP-sequencing datasets (settings for 300 bp, $p < 1 \times 10^{-5}$) (Liu et al., 2016 GEO accession number GSE73952, Fursova et al., 2021 GEO accession number GSE161996) between the histone mark ChIP and input libraries. The three biological replicates each for Smchd1wt and Smchd1matΔ were merged, and peaks were called against the MACS peaks from the publicly available data. using the feature probe generator function in SeqMonk (±2.5kb). Probes were quantitated using the read based quantitation option, normalising to library size and log2 transforming the read count. For CUT&RUN data at days 2, 2.5 and 3, MACS2 peaks were called between all samples and all inputs for each antibody, for browser track normalisation purposes. Differential peaks between samples using the csaw package v1.24.3 were called with default parameters.

Data quality

Data quality was assessed using FastQC v0.11.8. In addition, we compared our data in mESC to previously published data in mESC found at GEO accession number GSE73952, 42 GEO accession number GSE161996, and found high correlation, shown in the figures. For quality control during differentiation of mESC we analysed whether the H3K27me3 or H2AK119ub enriched regions over the Hox genes altered over time, as expected. This quality control is shown in the figures.

Software

SeqMonk v1.47.1, v1.47.2 or v1.48.0 was used and the feature probe generator function in SeqMonk (±2.5kb) was used to quantitate over probes (MACS peaks from public data), using the read based quantitation option, normalising to library size and log2 transforming the read count. Csaw v1.24.3 was used to call differential peaks between samples.
